# Selective Mass Accumulation at the Metal–Polymer Bridging Interface for Efficient Nitrate Electroreduction to Ammonia and Zn-Nitrate Batteries

**DOI:** 10.1021/jacs.5c00400

**Published:** 2025-06-11

**Authors:** Guojie Chao, Wei Zong, Jiexin Zhu, Haifeng Wang, Kaibin Chu, Hele Guo, Jian Wang, Yuhang Dai, Xuan Gao, Longxiang Liu, Fei Guo, Ivan P. Parkin, Wei Luo, Paul R. Shearing, Longsheng Zhang, Guanjie He, Tianxi Liu

**Affiliations:** † Key Laboratory of Synthetic and Biological Colloids, Ministry of Education, School of Chemical and Material Engineering, International Joint Research Laboratory for Nano Energy Composites, 66374 Jiangnan University, Wuxi 214122, P. R. China; ‡ Department of Engineering Science, 6396 University of Oxford, Parks Road, Oxford OX1 3PJ, U.K.; § Christopher Ingold Laboratory, Department of Chemistry, 4919 University College London, 20 Gordon Street, London WC1H 0AJ, U.K.; ∥ Department of Mechanical and Industrial Engineering, 7938 University of Toronto, Toronto, Ontario M5S 3G8, Canada; ⊥ State Key Laboratory for Modification of Chemical Fibers and Polymer Materials & College of Materials Science and Engineering, 12475 Donghua University, Shanghai 201620, China

## Abstract

The electrochemical conversion of nitrate (NO_3_^–^), a common nitrogen source in industrial wastewater and contaminated groundwater, into ammonia (NH_3_), signifies an approach to wastewater treatment and NH_3_ production. Nevertheless, its selectivity and activity at low NO_3_^–^ concentrations and industrial current densities are constrained by limited mass transfer around the electrode. Here, we report a metal–polymer bridging interface constructed by anchoring Cu/Cu_2_O nanoparticles onto a two-dimensional (2D) Cu-based benzene dicarboxylate (CuBDC) coordination polymer via in situ electroreduction (denoted as E-CuBDC). This interface weakens the electrostatic repulsion and regulates the distribution/migration of NO_3_^–^ and H_2_O, creating a Janus NO_3_^–^-rich and H_2_O-poor domain near the catalyst surface. Operando characterizations and theoretical simulations indicate that the metal–polymer bridging interface selectively accumulates NO_3_^–^ and reduces the energy barrier toward the reduction of *NH_2_OH to *NH_2_, overcoming the mass transfer limitations at a low NO_3_^–^ concentration. E-CuBDC exhibits a high Faradaic efficiency (FE) of over 90% across wide NO_3_^–^ concentrations (7.1–100 mM NO_3_^–^) and high applied voltages. Additionally, it achieved stable NH_3_ production over 100 h at ampere-level current densities. When applied in a Zn–NO_3_^–^ system, this newly developed E-CuBDC catalyst demonstrates an outstanding power density and FE for NH_3_ production, showcasing its great potential for large-scale electrochemical conversion and storage systems. This study presents a generalizable strategy for constructing metal–polymer interfaces to regulate interfacial mass transport.

## Graphical abstract

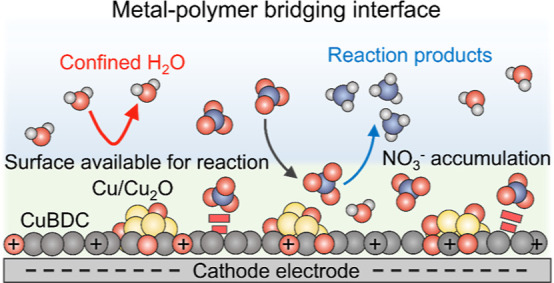

## Introduction

Ammonia (NH_3_) is one of the most widely utilized fundamental chemicals across numerous industries, including the fields of textiles, agriculture, and plastics, serving as the high-energy-density carrier for renewable hydrogen storage and utilization.^1–4^ Compared to chemically inert nitrogen (N_2_), NH_3_ is a more reactive and readily utilizable nitrogen source, thereby increasing the economic viability and practical applicability of nitrogen-based processes.^5,6^ In recent years, industrial-scale NH_3_ production by the Haber–Bosch method has succeeded in providing affordable fertilizers for supporting the NH_3_ industry and feeding the population. However, the energy-intensive NH_3_ synthesis method has led to high energy consumption and greenhouse gas emissions.^7–10^ Significant efforts have been directed toward developing innovative methods for green and sustainable NH_3_ electrosynthesis to reduce reliance on fossil fuels and mitigate environmental pollution. Electrochemical nitrate (NO_3_^–^) reduction reaction (NITRR) is an emerging technology that provides a more sustainable pathway process for NH_3_ production.^11–14^ Moreover, NO_3_^–^, commonly found as a water contaminant in nature owing to agricultural runoff and industrial wastewater discharge, is ubiquitous in the environment, leading to water pollution and human health hazards.^15^ NITRR not only serves as an operational tool for restoring the disrupted nitrogen cycle and expediting wastewater denitrification but also offers an eco-conscious and sustainable method for NH_3_ production.^16,17^ Nevertheless, the development of electrocatalysts with both high activity and high selectivity for NITRR poses a formidable challenge. Currently, Cu-based catalysts are regarded as promising candidates for NITRR due to their favorable ability to activate NO_3_^–^.^18–21^

For instance, Zhang et al.^18^ reported that CuO NWAs are beneficial to the formation of *NOH reaction intermediate and exhibit a high energy barrier of H_2_ formation, resulting in an enhanced selectivity for NO_3_^–^ reduction to NH_3_. Despite the progress made thus far, however, one of the persistent bottlenecks is the electrostatic repulsion occurring at the electrode–electrolyte interface, which hampers the mass transfer process of NO_3_^–^ and leads to a sharp decline in the concentration of NO_3_^–^ in the vicinity of electrode interfaces (Figure 1a).^22–24^ As a representative interface reaction, NITRR mainly comprises two fundamental stages: adsorption and catalysis, in which the initiation of NITRR relies on effective NO_3_^–^ adsorption, making it a crucial factor in surmounting the limitations associated with the low binding affinity of NO_3_^–^ reactants. Besides, the catalyst surface is predominantly occupied by water molecules, which favors the competing hydrogen evolution reaction (HER),^25–27^ leading to poor Faradaic efficiency (FE) and NH_3_ yield of NITRR (Figure 1b). This situation is further exacerbated by NITRR electrocatalysis in real wastewater treatment, including conditions of low concentration and industrial-scale current. To effectively and selectively promote NITRR, modulating the mass transfer near the electrode region could be a feasible approach to overcoming the challenges mentioned above.^28–30^ A microenvironment modulation that breaks the mass transfer restriction of NO_3_^–^ in the proximity of the catalyst surface might efficiently promote NITRR performance. Although the microenvironment is expected to play a significant role, assessing the extent of its impact on reaction processes remains highly challenging. Specifically, it is crucial to deeply investigate the relationship between the interface microenvironment and its influence on tailoring NITRR activity and selectivity.

**Figure 1:**
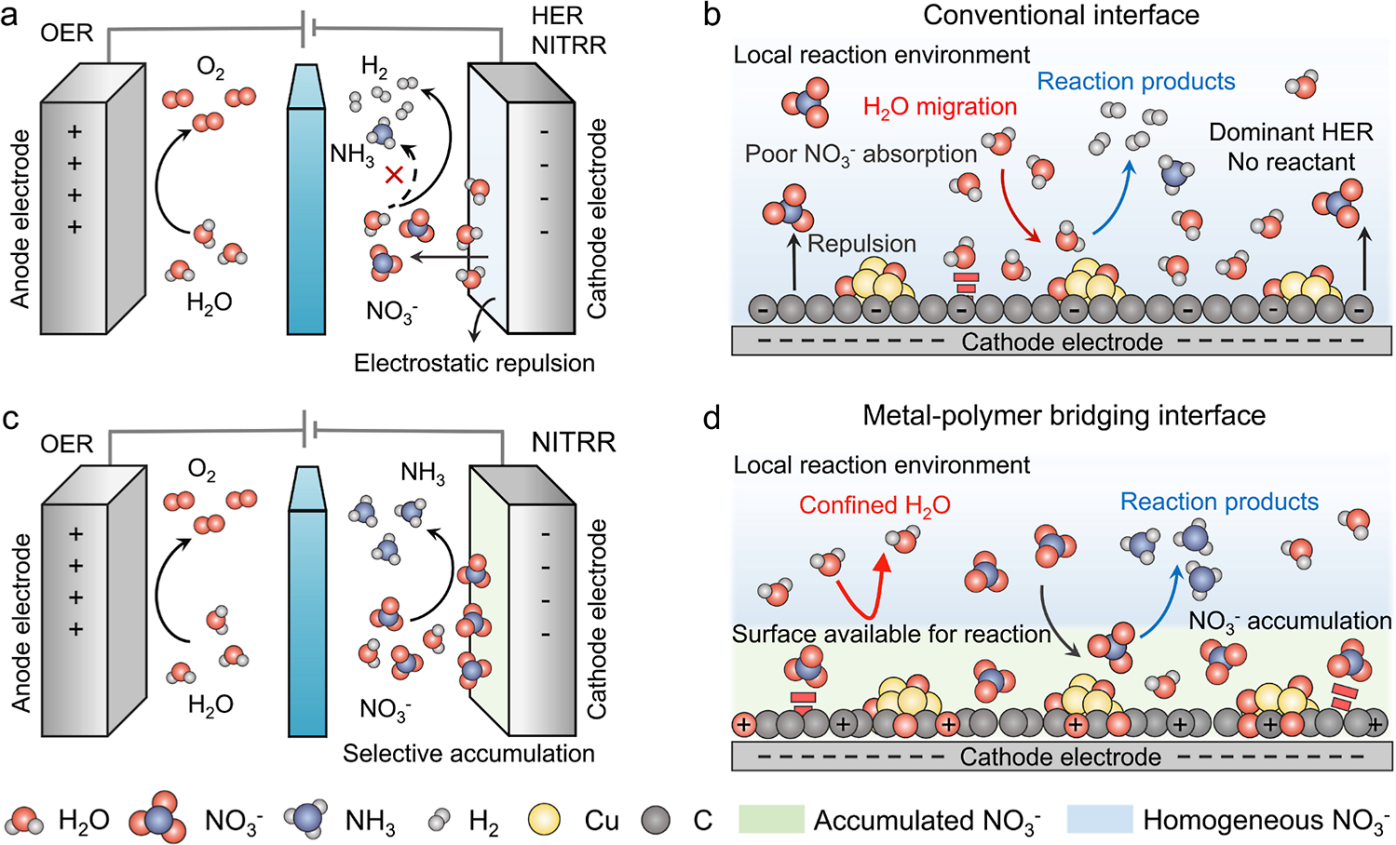
Schematics for the mass transport at the conventional and metal–polymer bridging interfaces. (a,b) Schematics illustrating the unregulated H_2_O and NO_3_^–^ mass transport at the conventional interface in the OER||NITRR electrolyzer. Random H_2_O migration and NO_3_^–^ expulsion occur in the local reaction environment near the cathode electrode, where electrostatic repulsion inhibits the access of NO_3_^–^ to the catalyst surface, favoring the HER over NITRR. (c,d) Schematics illustrating the controlled H_2_O and NO_3_^–^ mass transport at the metal–polymer bridging interface in the OER||NITRR electrolyzer. Confined H_2_O migration and NO_3_^–^ accumulation occur in the local reaction environment near the cathode electrode, favoring the NITRR over HER. Red ball represents the O atom, gray ball represents the H atom, dark gray ball represents the C atom, and dark blue ball represents the N atom. HER signifies the hydrogen evolution reaction, and OER signifies the oxygen evolution reaction.

In this work, we report a Janus metal–polymer bridging interface that enriches NO_3_^–^ and blocks H_2_O, enabling efficient electrochemical NH_3_ production from NO_3_^–^ across wide concentrations and at ampere-level current densities. This Janus interface is constructed by bridging Cu/Cu_2_O nanoparticles (NPs) onto a Cu-based benzene dicarboxylate (CuBDC) coordination polymer via an in situ electroreduction method. For the as-formed catalyst (denoted as E-CuBDC), a series of operando characterizations combined with theoretical simulations reveals that the metal–polymer bridging interface weakens electrostatic repulsion near the catalyst surface, thereby facilitating NO_3_^–^ mass transfer and confining H_2_O accessibility. Moreover, the metal–polymer interface enhances the adsorption of NO_3_^–^ and reduces the energy barrier of the step (*NH_2_OH to *NH_2_). The regulated distribution and migration of NO_3_^–^ and H_2_O create a NO_3_^–^-rich and H_2_O-poor domain near the catalyst surface. This affords the catalyst a kinetically favorable local environment for promoting NITRR and alleviating HER (Figure 1c,d). As a result, E-CuBDC achieves an exceptional FE over 90% across the NITRR electrocatalysis with NO_3_^–^ concentrations ranging from 7.1 to 100 mM and a high NH_3_ yield rate of 77.9 mg h^–1^ cm^–2^ at a current density of ∼1.2 A cm^–2^ in a flow cell. The performance evaluation in the Zn–NO_3_^–^ cell demonstrates a power density of 17.9 mW cm^–2^ and a FE of 82.2% for NH_3_ production.

## Results and Discussion

To explore the feasibility of the metal–polymer bridging interface design, we first synthesized CuBDC via an interfacial reaction method at room temperature (Figure S1). To modulate the catalyst structure, various CuBDC-*x* samples (denoted as CuBDC-1, CuBDC, and CuBDC-2) were synthesized through adjusting the molar ratios (1:1, 2:1, and 4:1) of copper acetate and 1,4-benzenedicarboxylic acid (TA). The catalyst with a balanced Cu/TA molar ratio of 2:1 was designated as CuBDC and served as a reference. The coordination bonds between the inorganic nodes and organic linker in CuBDC were first characterized by Fourier transform infrared (FTIR) spectroscopy. Two peaks of −COO– are observed at 1570 and 1397 cm^–1^ for TA and CuBDC-*x* samples, which were attributed to the asymmetric and symmetric vibrations of the −COO– group (Figure S2).^31^ A typical C–O–Cu stretching vibration peak at around 1091 cm^–1^ is observed. In sharp contrast to TA, the carbonyl peak associated with the protonated carboxyl group disappears in CuBDC-*x* samples within the range of 1710–1760 cm^–1^, indicative of coordination between Cu^2+^ and the carboxyl group. The crystalline structures of CuBDC-*x* samples were further validated by X-ray diffraction (XRD) and transmission electron microscopy (TEM). Indeed, Figure S3 shows that different CuBDC-*x* samples display similar diffraction peaks to those reported in the previous literature studies,^32,33^ indicating the preparation of crystalline CuBDC-*x*. As shown in Figure S4 from TEM images, CuBDC exhibits a well-defined 2D flaky structure with discernible lattice fringes, confirming its crystallinity. The CuBDC-*x* samples were utilized as catalysts following an electrochemical activation approach, which involved electrochemically reducing CuBDC in a 1 M KOH–0.1 M NO_3_^–^-N electrolyte to produce the E-CuBDC catalyst. Then, a series of characterizations were employed to elucidate the structural evolution and reaction mechanism of E-CuBDC. As displayed in Figures 2a and S5a, the TEM images of E-CuBDC demonstrate the uniform distribution of small bright clusters, each with a diameter of about 4–8 nm on the CuBDC surface, which arises from the electrochemical reduction of the Cu–O nodes in CuBDC. In the high-resolution TEM image of E-CuBDC (Figures 2b and S5b), twin lattice fringes with spacings of 0.206 and 0.245 nm were observed, corresponding to the Cu (111) and Cu_2_O (111) planes. Moreover, the high-angle annular dark-field scanning TEM image, coupled with X-ray energy-dispersive spectroscopy mapping images, further validates the spatial overlap of C, O, and Cu elements, highlighting the coexistence of Cu/Cu_2_O NPs and CuBDC (Figure S6).

**Figure 2:**
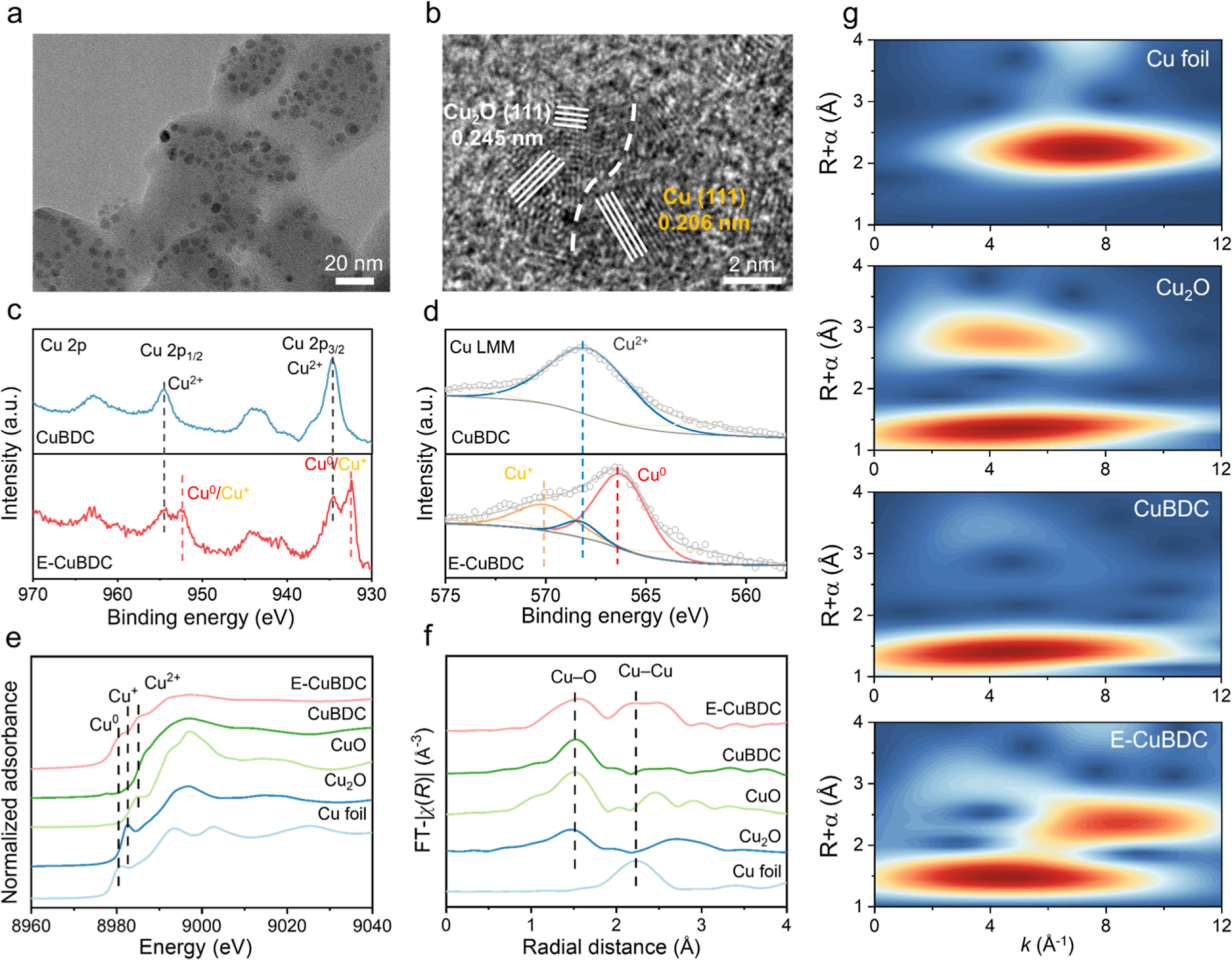
Materials synthesis and characterization. (a,b) Low- and high-magnification TEM images of E-CuBDC, respectively. (c) High-resolution Cu 2p X-ray photoelectron spectroscopy (XPS) and (d) Cu LMM Auger electron spectra of CuBDC and E-CuBDC. (e) Cu K-edge synchrotron radiation X-ray absorption near-edge structure (SR-XANES) and (f) synchrotron radiation extended X-ray absorption fine structure (SR-EXAFS) spectra of E-CuBDC, CuBDC, CuO, Cu_2_O, and Cu foil, respectively. (g) Wavelet transform analysis of E-CuBDC, CuBDC, Cu_2_O, and Cu foil.

This uniform distribution ensures effective interactions between the Cu/Cu_2_O NPs and CuBDC. Meanwhile, the formation of Cu and Cu_2_O species was verified through XRD (Figure S7) and Raman spectra (Figure S8).^34^ As shown in Figure S9, FTIR spectroscopy of E-CuBDC displays characteristic peaks at around 1570, 1397, and 1091 cm^–1^, corresponding to the antisymmetric and symmetric stretching vibration of the −COO– group and the C–O–Cu stretching vibration.^31,32^ The chemical components and valence states of the E-CuBDC and CuBDC were further investigated by XPS. Compared to CuBDC, two new peaks at 932.4 and 952.5 eV were observed in the high-resolution Cu 2p spectra of E-CuBDC (Figure 2c), which are associated with the Cu^0^/Cu^+^ species. Meanwhile, as shown in the Cu LMM Auger electron spectroscopy of E-CuBDC (Figure 2d), three characteristic peaks at binding energies of 570.1, 568.3, and 566.4 eV can be attributed to Cu^+^, Cu^2+^, and Cu^0^ species, respectively, confirming the formation of Cu/Cu_2_O NPs on the surface of CuBDC.^35–37^ Collectively, the above results demonstrate that the Cu/Cu_2_O NPs are introduced on the surface of CuBDC during the electrochemical reduction. The local structural characterization of Cu species was conducted through SR-XANES spectroscopy, utilizing CuO, Cu_2_O, and Cu foil as comparative references (Figure 2e). It can be found that the absorption near-edge position of CuBDC closely resembles that of the CuO reference, suggesting that its valence state is mainly Cu^2+^. In contrast, the SR-XANES Cu K-edge of E-CuBDC shifts toward low energy and displays characteristic peaks of Cu^+^ and Cu^0^ species. The SR-EXAFS spectra of CuBDC and E-CuBDC show an obvious peak at 1.5 Å assigned to the Cu–O scattering path (Figure 2f). Moreover, E-CuBDC exhibits a new peak located at ∼2.2 Å that originates from the scattering paths of the Cu–Cu bonds, demonstrating the formation of Cu^0^ in E-CuBDC.^38^ Furthermore, the structural reconstruction of the Cu species in E-CuBDC is corroborated by the wavelet transform analysis of SR-EXAFS spectra (Figure 2g). E-CuBDC shows an intensity maximum of about 4.5 Å^–1^ from the Cu–O bond, similar to that observed in CuBDC. The weak intensity at ∼8.3 Å^–1^ corresponding to the Cu–Cu bond is slightly higher than that of Cu foil (∼7.8 Å^–1^). The combined results above demonstrate that CuBDC underwent self-adaptive reconstruction during the NITRR process, which was transformed into E-CuBDC with superficially emended Cu/Cu_2_O NPs. The above result demonstrates that the metal–polymer interface was successfully constructed during the NITRR process (Figure S10 and Note S1).

To further seek experimental evidence for metal–polymer interface design, we evaluated the electrocatalytic performance of as-prepared catalysts toward NITRR in a customized H-cell under ambient conditions. As depicted in Figure S11, the linear sweep voltammetry (LSV) curves of E-CuBDC-*x* were recorded in a 1 M KOH solution with and without 0.1 M NO_3_^–^-N, respectively. The current density of the LSV curves shows a marked increase with the addition of NO_3_^–^ to the electrolyte, indicating the initiation of the NITRR process. In comparison to the commercial Cu and Cu_2_O samples (Figure 3a), E-CuBDC shows an enhanced geometrically normalized current density (*j*_NH_3__) of the NITRR process, and the highest *j*_NH_3__ of E-CuBDC indicates its superior catalytic activity for NITRR. The corresponding Tafel slope derived from the LSV curves for E-CuBDC was 341 mV dec^–1^, which was lower than other control samples (E-CuBDC-1 and E-CuBDC-2), Cu, and Cu_2_O catalysts (Figures S12 and S13). These above results imply that E-CuBDC exhibits improved reaction kinetics at the electrolyte–catalyst interface during the NITRR process. The NITRR performance was then determined by chronoamperometry (*i*–*t*) tests at various potentials ranging from −0.25 to −0.75 V vs reversible hydrogen electrode (RHE), as shown in Figure S14. Detection of NH_3_ and NO_2_^–^ production in electrolytes was carried out using an ultraviolet–visible spectrophotometer (Figure S15). As illustrated in Figure 3b,c, the NH_3_ yield rate of E-CuBDC progressively increased, and the corresponding NH_3_ Faradaic efficiency (FE_NH_3__) shows a volcanic shape. E-CuBDC reached a maximum of 96.6% at −0.65 V vs RHE, with an NH_3_ yield rate of 15.7 mg h^–1^ cm^–2^, in which the NH_3_ yield rate was approximately 1.5 times and 2.8 times than that of Cu_2_O and Cu reference, respectively. It was observed that both more positive and more negative potentials lead to a decrease in FE_NH_3__. This decline is attributed to two distinct factors: at more positive potentials, the insufficient supply of *H results in a less efficient *NO_2_ deoxygenation–hydrogenation process, while at more negative potentials, intensified competition from the HER process becomes dominant. Moreover, the continuous increase in NH_3_ yield rate is attributed to the abundant generation of *H, which promotes the hydrogenation steps toward NH_3_ production.^13,39^ In contrast, E-CuBDC can maintain higher FE_NH_3__ values at more negative potentials, such as 86.8% for E-CuBDC, 57.2% for Cu_2_O, and 56.5% for Cu at −0.75 V vs RHE.

**Figure 3:**
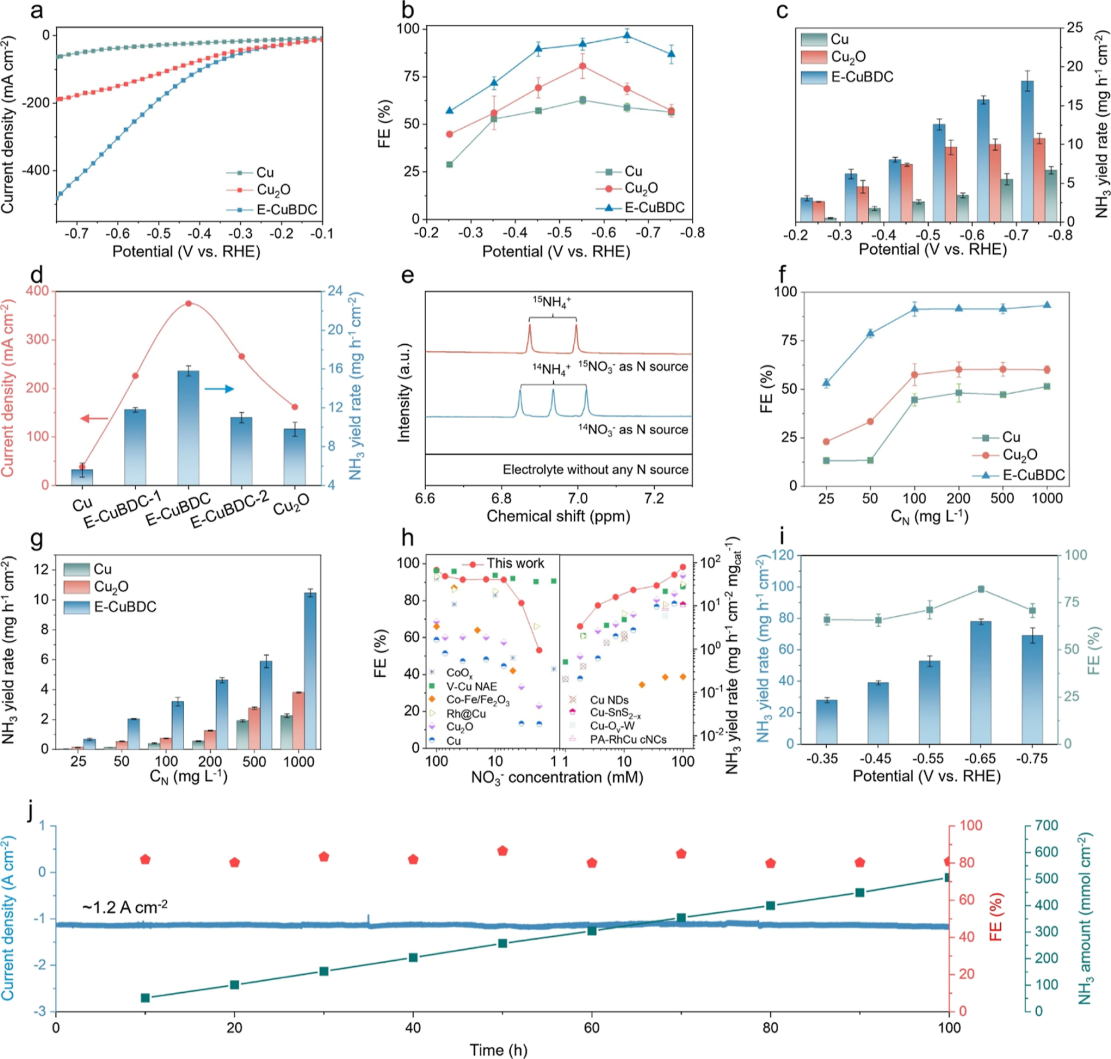
Electrochemical NITRR performance. (a) LSV curves of E-CuBDC, Cu_2_O, and Cu. (b,c) FE_NH_3__ and NH_3_ yield rate of E-CuBDC, Cu_2_O, and Cu at various potentials, respectively. (d) *j*_NH_3__ and NH_3_ yield rate for E-CuBDC-*x* with different Cu/TA ratios, Cu_2_O, and Cu. (e) ^1^H NMR spectra for the N-isotope labeling experiments of E-CuBDC. (f,g) FE_NH_3__ and NH_3_ yield rate of E-CuBDC with various NO_3_^–^-N concentrations from 25 to 1000 mg L^–1^, respectively. (h) Comparison of the NITRR performance between E-CuBDC and other previously reported electrocatalysts. (i) NH_3_ yield rate and FE_NH_3__ of E-CuBDC@Ni foam at various potentials. (j) Total NH_3_ amount and FE_NH_3__ of E-CuBDC@Ni foam in the stability experiment at −0.65 V vs RHE.

To elucidate the effect of Cu content on the NITRR performance, E-CuBDC-*x* catalysts with varying Cu/TA ratios were synthesized and tested. As shown in Figure 3d, with the increase of Cu/TA ratios, both *j*_NH_3__ and NH_3_ yield rates display noteworthy enhancements. A distinctive volcano-shaped trend is observed between the *j*_NH_3__ and the NH_3_ yield rate, reaching its peak on E-CuBDC, with the highest values of 364 mA cm^–2^ and 15.7 mg h^–1^ cm^–2^. This exceeded those of E-CuBDC-1 (i.e., 226 mA cm^–2^ and 11.7 mg h^–1^ cm^–2^) and E-CuBDC-2 catalysts (i.e., 266 mA cm^–2^ and 11.0 mg h^–1^ cm^–2^), as depicted in Figure S17. The *j*_NH_3__ and NH_3_ yield rates declined dramatically when the Cu/TA ratios exceeded 2. Therefore, maintaining a balanced Cu/TA ratio is crucial for sustaining the optimal catalytic performance in ammonia production. Furthermore, the electrochemically active surface area (ECSA) was measured in the nonfaradaic reaction range. E-CuBDC shows higher ECSA values than the other E-CuBDC-*x*, Cu, and Cu_2_O owing to the increased electro-active surface area. This corroborates its highest intrinsic activity for NH_3_ synthesis (Figures S18 and S19). Isotope labeling experiments were also conducted to confirm the nitrogen source of synthesized NH_4_^+^. Figure 3e presents the ^1^H NMR spectra of the electrolyte following NITRR tests, labeled with the ^15^N isotope, wherein characteristic double peaks of ^15^NH_4_^+^ at δ = 6.87 and 6.99 ppm were observed. These results suggest that produced NH_3_ originates entirely from NO_3_^–^ electroreduction.

To research the practicability potential, a series of experiments were performed. Considering the variability of the NO_3_^–^ levels in wastewater at different scenarios, it is imperative for the catalyst to demonstrate robust performance across a broad range of NO_3_^–^ concentrations. Therefore, we simulated the degradation of waste streams (e.g., textile and industrial wastewater) with NO_3_^–^-N concentrations ranging from 25 to 1000 NO_3_^–^-N mg L^–1^. As demonstrated in Figure 3f,g, E-CuBDC exhibits a FE_NH_3__ of 91.3% with a NO_3_^–^-N concentration of 100 mg L^–1^ (7.1 mM NO_3_^–^ concentration) and maintains FE_NH_3__ over 90% in the NO_3_^–^-N concentrations range from 100 to 1000 NO_3_^–^-N mg L^–1^. In contrast, Cu and Cu_2_O consistently lagged behind E-CuBDC in the NH_3_ yield rate and FE_NH_3__ across a wide range of concentrations. E-CuBDC exhibited a comparable NH_3_ yield rate and FE_NH_3__ at a NO_3_^–^-N concentration of 25 mg L^–1^, almost equivalent to the performance of Cu and Cu_2_O at a NO_3_^–^-N concentration of 100 mg L^–1^. This distinction validates the effect of the Cu/Cu_2_O–CuBDC interface in promoting NO_3_^–^ mass transfer, especially in low concentrations of NO_3_^–^. As presented in Figure 3h, the NH_3_ yield rate and the FE_NH_3__ of E-CuBDC are on par with those of the other reported catalysts for NITRR within the NO_3_^–^ concentration range of 25–1400 NO_3_^–^-N mg L^–1^ (1.78–100 mM NO_3_^–^ concentration, Tables S1 and S2). Meanwhile, under higher NO_3_^–^ concentrations (above 0.1 M) and neutral conditions, E-CuBDC still maintains high NH_3_ production with FE_NH_3__ over 90% (Figures S20 and S21).

The stability at industrially relevant current densities is a key requisite for practical applications. Further, we prepared working electrodes by spreading E-CuBDC ink on Ni foam (E-CuBDC@Ni foam) to simulate industrial applications, given the relatively inert activity of Ni for the NITRR. The Ni foam was commonly selected as a self-supporting substrate for nanostructured electrocatalysts due to its cost-effectiveness and superior electrical conductivity. In the illustrated LSV curves within the potential range from −0.35 to −0.75 V, E-CuBDC exhibits an ampere-level current density markedly surpassing that of the Ni foam (Figures S22 and S23). This indicates Ni foam exhibits no discernible impact on the NITRR process, aligning with previously reported findings.^40–43^ Notably, E-CuBDC achieves a high NH_3_ yield rate of 77.9 mg h^–1^ cm^–2^, with the highest FE_NH_3__ of 82.1% at −0.65 V vs RHE (Figure 3i). The stability test with continuous electrolyte flow demonstrated sustained current density at ∼1.2 A cm^–2^ over 100 h (Figure 3j). These results demonstrated the excellent performance of E-CuBDC across a broad range of NO_3_^–^ concentrations and ampere-level currents, providing a promising avenue for addressing the intricate challenges posed by NO_3_^–^-containing wastewater in industrial applications.

To study the function of CuBDC at the metal–polymer interface, we examined the NO_3_^–^ management by conducting molecular dynamics simulations to study the distribution/migration of NO_3_^–^ near the Cu_2_O and CuBDC surface. The initial configurations in the simulation boxes were established by randomly distributing the NO_3_^–^ and H_2_O with Cu_2_O and the CuBDC layer. In the constructed model, the direction from left to right is defined as the *Z*-direction (Figure 4a,b). Of note is that there is a higher NO_3_^–^ concentration near the surface of the CuBDC model compared to the Cu_2_O model (Figure 4c). This indicates that CuBDC can offer anchoring sites to trigger the interfacial accumulation of NO_3_^–^. Additionally, we found that CuBDC, functioning as a support, can modulate the surface potential. As depicted in Figure S24, the surface zeta potential of CuBDC is determined to be 3.95 ± 0.67 mV, compared with those of Cu_2_O (−12.16 ± 1.15 mV) and Cu (−16.07 ± 0.51 mV). The relatively positive surface potential of CuBDC is conducive to the adsorption and enrichment of NO_3_^–^. Hence, the construction of the NITRR catalysts with a more positive surface potential may facilitate overcoming poor mass transfer and low-concentration gradients in the vicinity of the catalyst domain. Furthermore, we tracked changes in NO_3_^–^ and H_2_O concentrations around the catalyst surface of Cu/Cu_2_O and E-CuBDC after variation of the surface potential. Two models were constructed based on experimental observations, featuring Cu/Cu_2_O NPs decorated onto the surface of the CuBDC (Figure S25). Finite-element simulations were employed by using the transport of diluted species modules.

**Figure 4:**
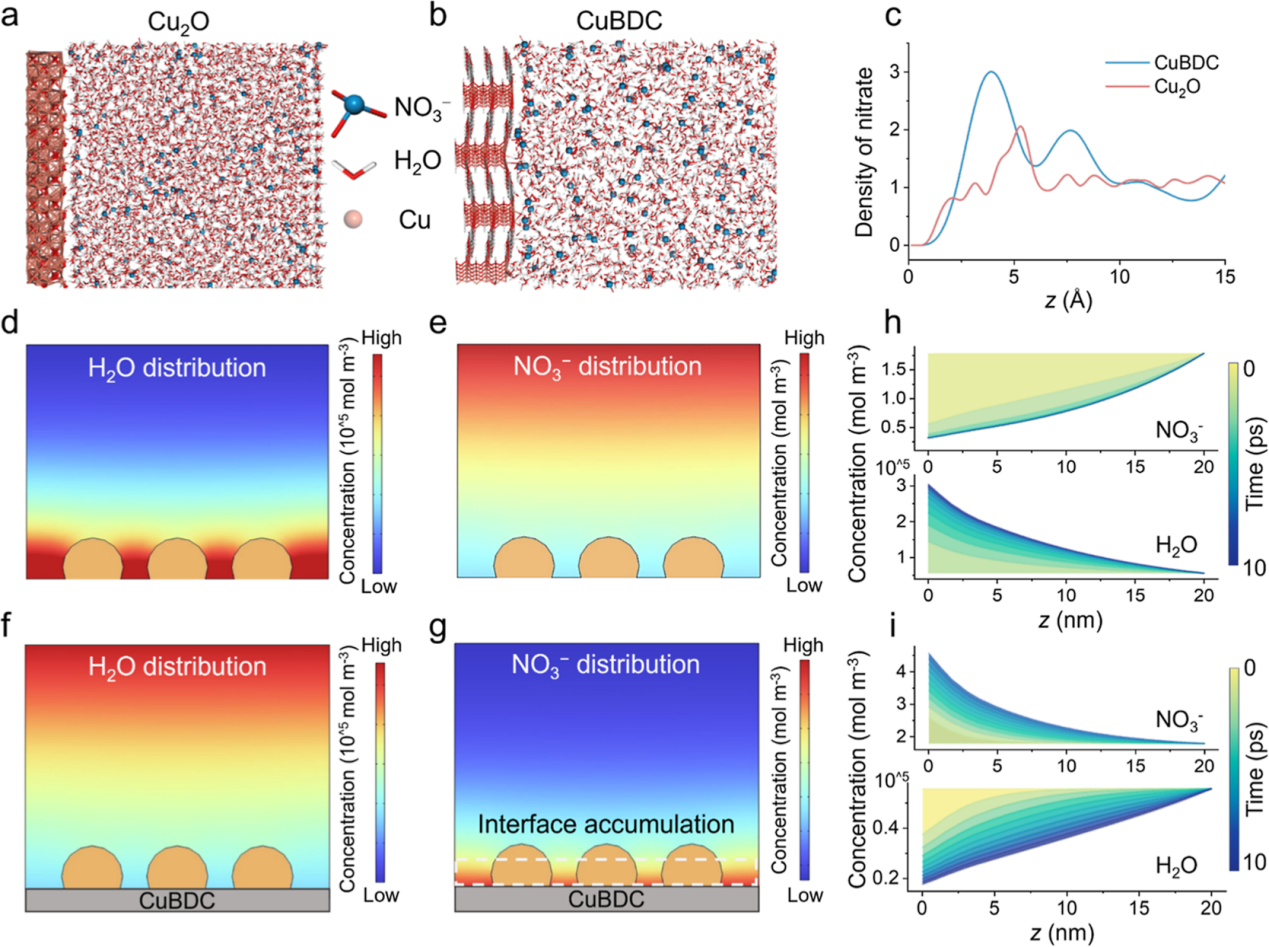
Molecular dynamics and COMSOL Multiphysics simulations. (a,b) Snapshots of the interfacial structures of Cu_2_O and CuBDC models from molecular dynamics simulations, respectively. (c) Corresponding interfacial accumulated density of NO_3_^–^ for the CuBDC and Cu_2_O models. (d,e) Distributions of H_2_O and NO_3_^–^ at 10 ps for the Cu_2_O model. (f,g) Distributions of H_2_O and NO_3_^–^ for the E-CuBDC model at 10 ps. (h,i) The concentrations of H_2_O and NO_3_^–^ as a function of distance from the catalyst surface for Cu/Cu_2_O and E-CuBDC at various times.

Figure 4d,e illustrates the evolution of NO_3_^–^ and H_2_O concentrations in the Cu/Cu_2_O model without the CuBDC support in the 3D simulation domain. It can be found that NO_3_^–^ migrates away from the surface of Cu/Cu_2_O while massive water molecules accumulate, which can raise the competitive HER and decrease the selectivity toward NITRR. In contrast, a substantial interface accumulation of NO_3_^–^ is observed, whereas H_2_O molecules are nearly excluded from the surface of Cu/Cu_2_O with the presence of the CuBDC support (Figure 4f,g). To better monitor the diffusion processes, we tracked changes in the NO_3_^–^ and H_2_O concentrations around the catalyst surface of Cu/Cu_2_O and E-CuBDC over time from 0 to 10 ps. As depicted in Figure 4h, the NO_3_^–^ concentration rapidly decreases after 2 ps in the Cu/Cu_2_O model, accompanied by a gradual increase in H_2_O concentration. In sharp contrast, the NO_3_^–^ concentration gradually increased, and the H_2_O concentration declined on the E-CuBDC surface with time from 0 to 10 ps (Figures 4i and S26). All of these results illustrate that the Cu/Cu_2_O-CuBDC interface in E-CuBDC creates a unique water-deficient and NO_3_^–^-rich interface, suppressing the HER process and providing abundant unoccupied active sites for the NITRR process.

To gain mechanistic insights into how the metal–polymer interface regulates interfacial mass transport, we employed in situ attenuated total reflection surface-enhanced infrared absorption spectroscopy (ATR-SEIRAS). This technique also enables identification of reaction intermediates and provides information on the surface environment during the NITRR process. As shown in the ATR-SEIRAS spectra of E-CuBDC and Cu_2_O (Figure 5a,b), the characteristic peak at 1384 and 1349 cm^–1^ is attributed to the N–O symmetric and asymmetric stretching vibration of *NO_3_^–^,^44^ attributing to the consumption process of NO_3_^–^. The *NO_3_^–^ peak of E-CuBDC declines much more than that of Cu_2_O, indicating a higher concentration of *NO_3_^–^ in the vicinity of E-CuBDC.

**Figure 5:**
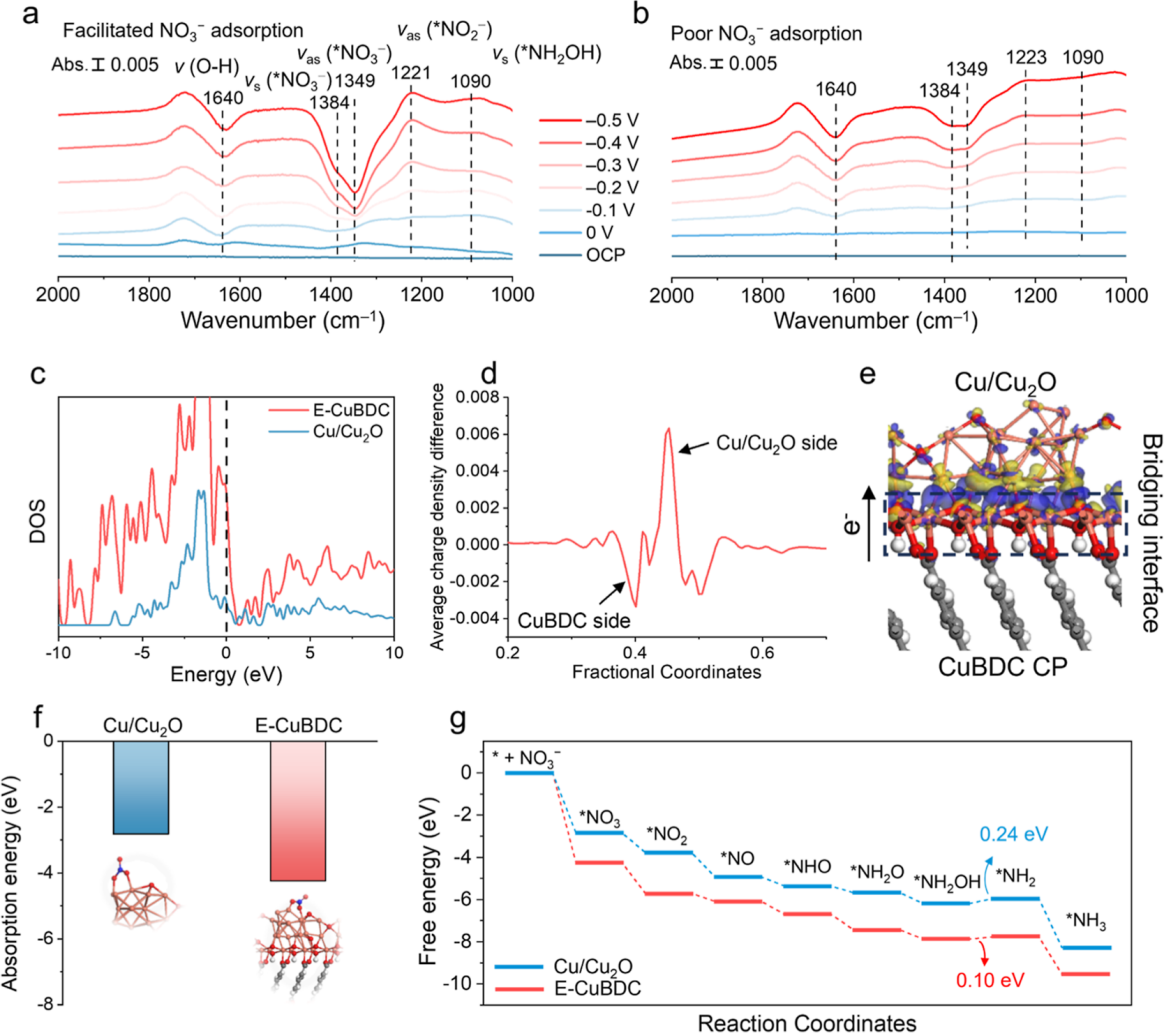
In situ ATR-SEIRAS characterization and density functional theory (DFT) calculations. (a,b) In situ ATR-SEIRAS spectra under NITRR operation for the E-CuBDC and Cu_2_O catalysts, respectively. (c) Density of states (DOS) for the E-CuBDC and Cu/Cu_2_O models. (d,e) Plane-averaged charge density difference at the Cu/Cu_2_O-CuBDC bridging interface in the E-CuBDC model. (f) Calculated NO_3_^–^ adsorption energies on the Cu/Cu_2_O and E-CuBDC models. (g) Free-energy profiles of NITRR on the Cu/Cu_2_O and E-CuBDC models with uphill energy barriers of 0.24 and 0.10 eV at the rate-determining step (*NH_2_OH → *NH_2_).

The upward band observed at around 1221 cm^–1^ is associated with the −N–O– antisymmetric stretching vibration of *NO_2_^–^,^45^ implying the NO_2_^–^ formation from the NO_3_^–^ reduction. Upon the potential shifting negatively to −0.5 V, another upward absorption band emerges at around 1090 cm^–1^, attributed to the stretching vibration of hydroxylamine (NH_2_OH),^41,45^ a crucial intermediate in NH_3_ formation. The enhanced downward band at roughly 1640 cm^–1^ is linked to water electrolysis,^46^ related to hydrogen production during the hydrodeoxidation of NO_3_^–^.^47^ It deserves to note the start and enhancement of *NO_3_^–^ band consumption on E-CuBDC at −0.1 V vs RHE, which were 200 mV more negative compared to pure Cu_2_O. This indicates significantly increased adsorption of NO_3_^–^ on the surface of E-CuBDC, aligning with the results obtained from molecular dynamics and finite-element simulations. Hence, the Janus metal–polymer interface in E-CuBDC facilitates the migration and accumulation of NO_3_^–^, leading to the formation of a NO_3_^–^-rich domain near the catalyst surface. This provides a kinetically favorable local environment for the NITRR process. Besides, with the negative shift in working potentials, the band intensity of NO_2_^–^ decreases, accompanied by an enhancement in the band intensity of NH_2_OH generation, a distinction not observed with pure Cu_2_O. The emergence of NH_2_OH subsequent to NO_2_^–^ formation, along with the rise in NH_2_OH with the depletion of NO_2_^–^, implies that NH_2_OH arises through the hydrogenation of NO_2_^–^. Further, we carried out in situ differential electrochemical mass spectrometry (DEMS) to detect potential intermediates (Figure S27). With the extension of time, signals corresponding to *m*/*z* 46, 30, 31, 33, 16, and 17 were observed, which can be attributed to the reaction intermediates of NO_2_, NO, HNO, NH_2_OH, NH_2_, and NH_3_, respectively.^18^ Combined with the in situ ATR-SEIRAS and DEMS, we proposed a possible route during the NITRR process on E-CuBDC: NO_3_^–^ → *NO_3_ → *NO_2_ → *NO → *HNO → *NH_2_OH → *NH_2_ → *NH_3_ (Figure S28).

Furthermore, DFT calculations were further conducted. First, we constructed simplified models of Cu/Cu_2_O and E-CuBDC. As shown in Figure 5c, the DOS of E-CuBDC has a higher level near the Fermi level than that of Cu/Cu_2_O at the Fermi level, suggesting promoted electron transfer to E-CuBDC. The charge density difference shown in Figure 5d,e shows that electrons transfer from the CuBDC substrate to Cu/Cu_2_O, implying a negative and positive charge distribution on the Cu/Cu_2_O and CuBDC substrate, respectively. The positive CuBDC substrate can form a boosted interfacial electric field, facilitating the absorption of anions such as NO_3_^–^ or NO_2_^–^ on the CuBDC surface. Moreover, the metal-centered Cu, featuring a less positive charge, functions as an electron reservoir capable of supplying and delivering electrons. The extra electrons localized near the Cu sites can greatly enhance the occupancy of the partially filled d-orbital, which can tailor the adsorption energy of NITRR intermediates, resulting in lowered energy barriers for intermediate formation. To further validate the above hypothesis, we introduced a NO_3_^–^ onto the surfaces of E-CuBDC and Cu/Cu_2_O (Figure 5f). The adsorption energy of NO_3_^–^ on E-CuBDC (−4.24 eV) is lower than that on Cu/Cu_2_O (−2.82 eV). This confirms a strong attractive effect of E-CuBDC for NO_3_^–^, thereby expediting the kinetics of NO_3_^–^ transfer for subsequent reduction processes on Cu/Cu_2_O, consistent with the findings of in situ ATR-SEIRAS. Based on the results offered by online DEMS and in situ ATR-SEIRAS, the NITRR process was investigated on E-CuBDC and Cu/Cu_2_O, involving *NO_3_, *NO_2_, *NO, *NHO, *NH_2_O, *NH_2_OH, and *NH_2_ intermediates, as depicted in Figures S29 and S30. From the corresponding Gibbs free-energy profile, we identified that the rate-determining step is step 7 (*NH_2_OH → *NH_2_), which shows a lower energy barrier of 0.10 eV compared to that of Cu/Cu_2_O of 0.24 eV (Figure 5g). These theoretical calculations, taken together, suggest that the improved NITRR performance of E-CuBDC may arise from its accelerated reaction kinetics and thermodynamics.

Inspired by the superior performance of E-CuBDC for ammonia production, we proceeded to construct Zn–NO_3_^–^ batteries using Zn plates as the anode and E-CuBDC as the cathode (Figure 6a), which presents great potential for sustainable ammonia synthesis and power generation.^48–53^ As shown in Figure 6b, the open-circuit voltage (OCV) of the Zn–NO_3_^–^ battery using the E-CuBDC cathode is approximately 1.28 V vs Zn. The OCV remains stable for over 45 h, highlighting the battery’s ability to sustain a consistent voltage over an extended duration. During discharge, the anodic dissolution of Zn drives the NITRR at the E-CuBDC cathode. The Zn–NO_3_^–^ battery exhibits a stable discharge profile characterized by a progressively decreasing potential coupled with a continuously increasing output current. This behavior remains consistent across measurements conducted at various discharge current densities, indicating its superior discharge performance (Figure 6c). To validate the Zn–NO_3_^–^ battery as a dual-output system capable of producing valuable chemicals and electricity, the NH_3_ yield rate and FE_NH_3__ were tested at increasing current densities (Figure 6d). Note that at current densities up to 40 mA cm^–2^, the Zn–NO_3_^–^ battery shows a high FE_NH_3__ of 82.2%, yielding a NH_3_ yield rate of 3.4 mg h^–1^ cm^–2^. The polarization profiles of the Zn–NO_3_^–^ battery with an E-CuBDC cathode during discharging are presented (Figure 6e). The Zn–NO_3_^–^ battery using the E-CuBDC cathode achieves a maximum power density of 17.9 mW cm^–2^, exceeding those of Cu_2_O (8.1 mW cm^–2^) and Cu (5.4 mW cm^–2^), as present in Figure S31. We also evaluated the long-term stability of the Zn–NO_3_^–^ battery employing the E-CuBDC cathode. As shown in Figure S32, the Zn–NO_3_^–^ battery exhibits sustained voltage output for 140 h, underscoring the excellent stability of the system using the E-CuBDC cathode. In the realm of research for aqueous Zn-based batteries, this study stands out for its power density and durability, outperforming most reported Zn–nitrogen hybrid systems, including Zn–NO_3_^–^, Zn–NO_2_^–^, and Zn–N_2_/NO batteries (Figure 6f and Table S3).

**Figure 6:**
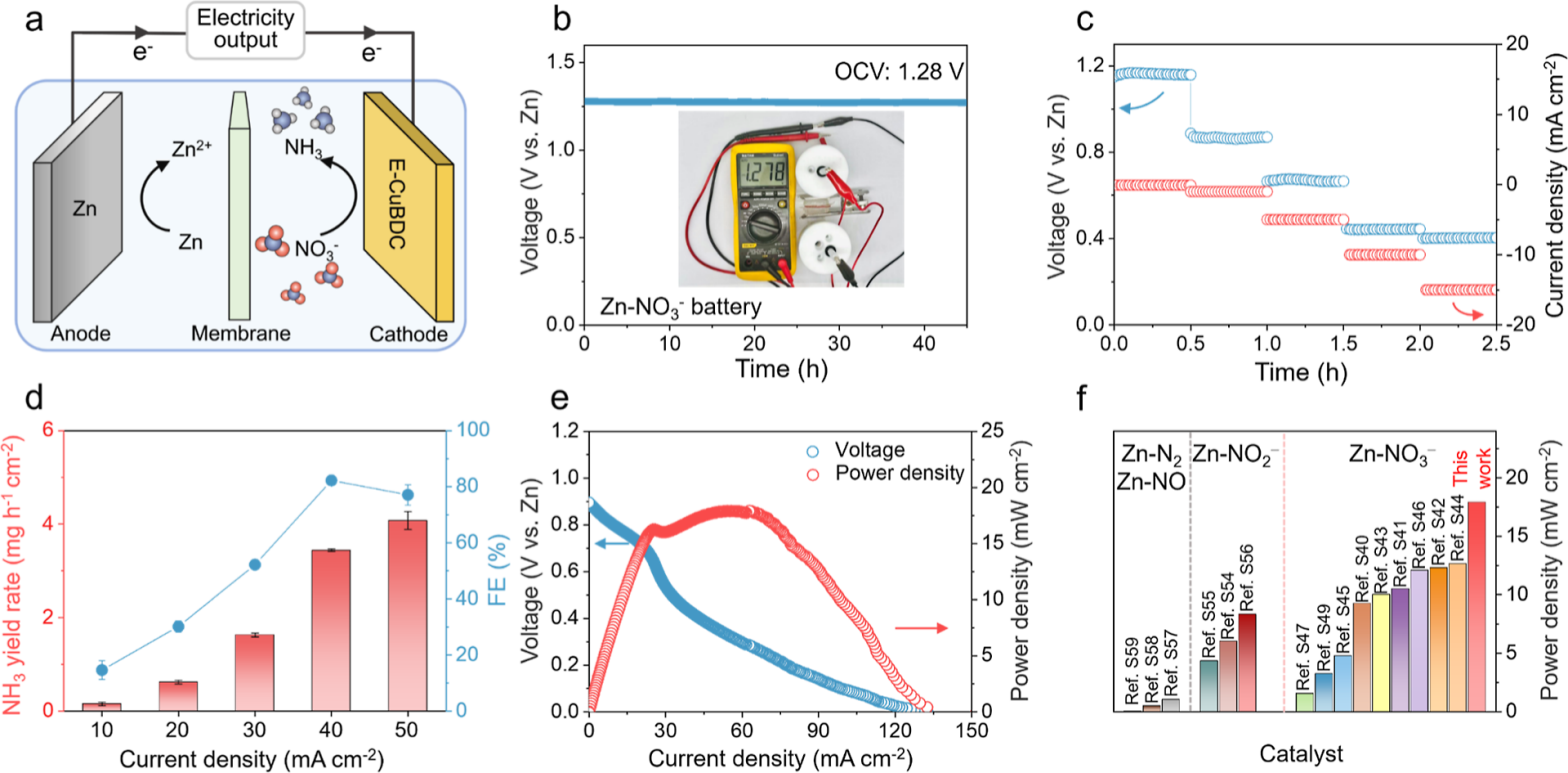
Zn–NO_3_^–^ battery performance. (a) Schematic illustration of the Zn–NO_3_^–^ battery assembled with the E-CuBDC cathode. (b) OCV curve of the Zn–NO_3_^–^ battery assembled with the E-CuBDC cathode. Inset of (b) shows the photograph of the OCV measurement of the Zn–NO_3_^–^ battery. (c) Rate capability of the Zn–NO_3_^–^ battery at different discharge current densities. (d) NH_3_ yield rate and FE of the Zn–NO_3_^–^ battery at different current densities. (e) Discharge curve and power density of the Zn–NO_3_^–^ battery. (f) Performance comparison between the Zn–NO_3_^–^ battery with the E-CuBDC cathode and other reported Zn-nitrogen hybrid systems, including Zn–NO_3_^–^, Zn–NO_2_^–^, and Zn–N_2_/NO batteries.

## Conclusion

In summary, we design a metal–polymer composite with Cu/Cu_2_O nanoparticles bridged on a 2D Cu-based benzene dicarboxylate by in situ electroreduction, which shows efficient and highly selective NH_3_ synthesis from electrochemical nitrate (NO_3_^–^) reduction reaction. Electrochemical reduction of NO_3_^–^ to NH_3_, instead of N_2_, enhances the economic and practical value of the process. NH_3_ serves as a crucial nitrogen source for agricultural fertilizers, a key chemical feedstock for various industrial applications, and a high-energy-density carrier for renewable hydrogen storage and utilization. The optimal E-CuBDC exhibits a FE_NH_3__ of 96.6% with a NH_3_ yield rate of 15.7 mg h^–1^ cm^–2^ at −0.65 V vs RHE. More significantly, E-CuBDC achieves high FE_NH_3__ over 90% across wide NO_3_^–^ concentrations (100–1400 NO_3_^–^-N mg L^–1^) and under high applied voltages. It maintains stable NH_3_ production over 100 h at around 1.2 A cm^–2^. The ATR-SEIRAS, finite-element simulations, and DFT calculations confirm that the constructed Cu/Cu_2_O-CuBDC Janus interface can induce synergistic modulation of facilitating the mass accumulation of NO_3_^–^ and restricting H_2_O accessibility at the electrode surface. Further, as demonstrated, the Zn–NO_3_^–^ battery using E-CuBDC as a cathode simultaneously generates electricity and produce NH_3_, with a power density of 17.9 mW cm^–2^ and a FE_NH_3__ of 82.2%. This study highlights an effective methodology of constructing high-performance catalysts with metal–polymer interfaces in efficient NH_3_ synthesis from NO_3_^–^ reduction electrocatalysis and Zn-based hybrid electrochemical energy storage systems.

## Supplementary Material



## Notes

Detailed experimental procedures, characterizations, computational methods, FTIR spectra, XRD patterns, TEM images, low- and high-magnification TEM images, high-angle annular dark-field scanning TEM image, Raman spectra, LSV curves, Tafel slopes, *i*–*t* curves, surface zeta potential, DEMS measurements (PDF)
