# Supplementary material for: Selective Mass Accumulation at the Metal–Polymer Bridging Interface for Efficient Nitrate Electroreduction to Ammonia and Zn-Nitrate Batteries
Source: J Am Chem Soc. 2025 Jun 11;147(25):21432–42. doi: 10.1021/jacs.5c00400 (PMC12203617; doi:10.1021/jacs.5c00400)
Supplement: Supplementary file 1 [file ja5c00400_si_001.pdf]

Supporting Information

Selective Mass Accumulation at Metal-Polymer Bridging Interface for Efficient Nitrate Electroreduction to Ammonia and Zn-Nitrate Batteries

Guojie Chao[a, $], *Wei Zong*[b, c, $, *], *Jiexin Zhu*[d, $], *Haifeng Wang*[e, $], *Kaibin Chu*[a], *Hele Guo*[a], *Jian Wang*[a], *Yuhang Dai*[b], *Xuan Gao*[b], *Longxiang Liu*[c], *Fei Guo*[c], *Ivan P. Parkin*[c], *Wei Luo*[e], *Paul R. Shearing*[b], *Longsheng Zhang*[a,*], *Guanjie He*[c,*], *and Tianxi Liu*[a,*]

a Key Laboratory of Synthetic and Biological Colloids, Ministry of Education, School of Chemical and Material Engineering, International Joint Research Laboratory for Nano Energy Composites, Jiangnan University, Wuxi 214122, P. R. China

b Department of Engineering Science, University of Oxford, Parks Road, Oxford OX1 3PJ, UK

c Christopher Ingold Laboratory, Department of Chemistry, University College London, 20 Gordon Street, London WC1H 0AJ, UK

d Department of Mechanical and Industrial Engineering, University of Toronto, Toronto, ON M5S 3G8, Canada

e State Key Laboratory for Modification of Chemical Fibers and Polymer Materials & College of Materials Science and Engineering, Donghua University, Shanghai 201620, China

$ These authors contributed equally to this work.

**Corresponding authors*

This file includes:

1. Materials and Methods

2. Supplementary Figures

3. Supplementary Tables

1. Materials and Methods

Materials

1,4-benzendicarboxylic acid; dimethylacetamide (TA), $CHCl_3$; $Cu(Ac)_2 \cdot H_2O$; sodium acetate (NaOAc), sodium hydroxide (NaOH), disodium hydrogen phosphate (Na_2HPO_4), sulfamic acid, sodium citrate, sodium hypochlorite, sodium nitrite ferricyanide, sulfamic acid (NH_2SO_3H), phosphoric acid (H_3PO_4), hydrochloric acid (HCl), N-(1-naphthyl) ethylenediamine dihydrochloride ($C_{12}H_{16}Cl_2N_2$), ethanol, and Nafion® (5 wt% in mixture of lower aliphatic alcohols and water) were purchased from Sinopharm Chemical Reagent Co., Ltd.

Preparation of CuBDC

Synthesis of Cu terephthalate (CuBDC) frameworks: The CuBDC was prepared via an interface reaction strategy. Firstly, the 1,4-benzendicarboxylic acid (TA) (24.9 mg) was dispersed in the mixture solution of dimethylacetamide (6 mL) and $CHCl_3$ (6 mL) under magnetic agitation. Meanwhile, 60 mg $Cu(Ac)_2 \cdot H_2O$ was dissolved in 6 mL deionized water by ultrasonic treatment. After, the $Cu(Ac)_2 \cdot H_2O$ aqueous solution was added to the above TA solution. Quickly, stratification occurs between the interface of the two solutions and the products were grown in an ambient environment. The synthesized sample was washed with copious amounts of deionized water and ethanol, collected by centrifuging, and then dried in a vacuum at 60 °C for 12 h. The obtained sample was named as CuBDC, with a molar ratio of 2 (Cu/TA: 2/1). By adjusting the Cu/TA ratio, CuBDC-1 and CuBDC-2 were further synthesized with molar ratios of 1/1 and 4/1, respectively. All other preparation conditions remained the same as those for CuBDC. The in-situ electroreduction activation of CuBDC was carried out by performing cyclic voltammetry (CV) tests until steady-state CV curves were achieved. The activation process was conducted at a scan rate of 20 mV s^{-1} over a potential range from 0 to -1.2 V in 1 M KOH aqueous solution containing 0.1 M KNO_3, yielding the material designated as E-CuBDC.

Characterization

The morphologies of samples were observed using scanning electron microscopy (SEM, JSM-7500F, JEOL) and transmission electron microscopy (TEM, FEI Talos F200x G2, FEI) with an energy-dispersive X-ray spectroscopy (EDS) detector. X-ray diffraction (XRD) patterns were recorded by using the X' Pert Pro X-ray diffractometer with Cu Kα radiation (λ = 0.1542 nm). Fourier transform infrared spectroscopy (FTIR) spectra were recorded on a Nicolet Nexus 870 instrument using the attenuated total reflection mode. X-ray photoelectron spectroscopy (XPS) measurements were conducted on a VG ESCALAB 220I-XL device. All XPS spectra were corrected with a C 1 s spectral line of 284.8 eV. Raman spectroscopy was tested on the Raman microscope (HORIBA Lab RAM HR Evolution) under an excitation of 532 nm laser light. The ultraviolet-visible (UV-Vis) absorbance spectra was measured on Beijing Purkinje General T700 new century spectrophotometer. X-ray absorption spectroscopy at the Cu K-edge was recorded using the transmission mode at the BL11B beamline station of the Shanghai Synchrotron Radiation Facility (SSRF). The XAS data was subsequently processed and analyzed with Athena and Artemis software, which are included in the standard IFEFFIT package. The specific surface area and pore size distribution were determined by collecting N_2 sorption/desorption isotherms at -196 °C using a QUADRASORB SI automated surface area and pore size analyzer (Quantachrome Corporation).

Electrochemical measurement

The electrochemical measurements were performed using an electrochemical working station (CHI660E, Shanghai, China) in an H-type electrolytic cell with a standard three-electrode system under ambient condition. The electrochemical tests were performed in 1 M KOH aqueous solution with or without 0.1 M KNO_3. Typically, the catalysts (10 mg) were added into a mixed solution of ethanol (0.94 mL) and Nafion solution (0.06 mL). Then, the solution was subjected to ultrasonication for 0.5 h to prepare homogeneous ink. Afterward, the working electrode was prepared by depositing the as-obtained catalyst ink (20 μL) onto the hydrophobic carbon paper (CP, TGP-H-60, Toray Carbon Paper), whose surface area was controlled with 1 cm^2. Then, linear sweep voltammetry (LSV) curves were measured in 1 M KOH aqueous solution with or

without 0.1 M KNO_3 at 10 mV s^{-1}. Chronoamperometry tests (*i-t*) were performed at various applied potentials for 2 h with a stirring rate of 400 rpm. The durability of the catalyst was measured by *i-t* tests. To further research the NITRR performance of E-CuBDC under low-concentration nitrate solution, *i-t* tests were performed at −0.65 V vs. RHE in the 1 M KOH electrolyte with various NO_3^-–N concentrations. Cyclic voltammetry (CV) curves used for determining the electrochemical double-layer capacitance (C_{dl}) were collected under conditions with no Faradaic processes occurring, which were conducted employing varying scanning rates of 20, 40, 60, 80, and 100 mV s^{-1}. All the current densities were obtained by normalizing current to the geometric surface area of the CP substrate.

To illustrate the industrial applicability of the obtained catalyst, we utilized chronopotentiometry test. The composition and configuration of the electrolytic cell remained consistent with the previous chronoamperometry test, except for an increased electrolytic cell volume of 80 ml. For electrode preparation, Ni foam (0.5 × 0.5 cm^2), decorated with 1 mg of catalysts, served as the working electrode. The *i-t* tests were conducted at various applied potentials for 0.5 h with a stirring rate of 400 rpm. To assess catalyst stability at high current density, Ni foam with E-CuBDC was tested at −0.65 V vs. RHE for 100 h. To mitigate the impact of nitrate concentration changes, a 5 L electrolyte container connected to the cathode via a pipe was prepared, and a peristaltic pump was introduced to facilitate mass transfer with a liquid circulation speed of 100 ml min^{-1}.

The potential presented in this work was converted to the V vs. RHE using the following equation:

$$E_{RHE} = E_{Ag/AgCl} + 0.059pH + E^0_{Ag/AgCl} \quad (1)$$

Determination of ammonia

UV-vis spectrophotometer was used to detect the ion concentration of pre- and post-test electrolytes after diluting to the appropriate concentration to match the range of calibration curves. The standard curve can be generated by examining the UV-Vis spectra of solutions with varying concentrations of NH_4^+. Typically, an appropriate

amount of electrolyte was taken out from the cathode chamber and diluted into appropriate concentration. 2 mL of salicylic acid solution (10 g salicylic acid and 10 g sodium citrate was first dissolved in 50 mL of water, followed by adding 55 mL of 2 M sodium hydroxide solution), 0.05 mL of 0.05 M sodium hypochlorite solution and 0.2 mL of 0.034 M sodium nitrite ferricyanide solution were added to the above diluted electrolyte. After the mixed electrolyte solution was kept in a dark condition for 2 h. The concentration-absorbance standard curve was calibrated with different concentrations of ammonium chloride standard solution.

Determination of nitrite ions (NO_2^-)

UV-vis spectrophotometer was used to quantify the NO_2^- concentration in the electrolyte before and after testing. First, to prepare the color reagent, 20 g of *p*-aminobenzenesulfonamide was added to a mixture of 250 mL deionized water and 50 mL phosphoric acid, followed by dissolution of 1 g *N*-(1-naphthyl)ethylenediamine dihydrochloride. The resulting solution was transferred to a 500 mL volumetric flask and diluted to volume with deionized water. Then, before UV-Vis measurement, the electrolyte was collected and diluted to the detection concentration range. Then, 0.1 mL of the color reagent was added to the diluted electrolyte. After thorough mixing and standing at room temperature for 30 minutes under ambient and dark conditions, the absorbance at a wavelength of 540 nm of the solution was recorded using UV-Vis spectrophotometry. The concentration–absorbance curve was calibrated using a series of standard potassium nitrite solutions with linear fitting prepared beforehand. NO_2^- product concentrations were calculated based on the tested absorbance and the standard curve.

Calculation of the ammonia yield and Faradaic efficiency (FE)

Calculation of the NH_3 yield and FE: the calculation equation is as follows:

$$\mathrm{Yield}_{\mathrm{NH_3}} = (c_{\mathrm{NH_3}} \times V)/(M_{\mathrm{NH_3}} \times t \times A) \quad (2)$$

where c_{NH_3} is the concentration of NH_3, V is the volume of electrolyte in the cathode compartment, M_{NH_3} is the molar mass of NH_3, t represents the time of the electrolysis process, A is the geometric area of working electrode.

$$\text{Faradaic efficiency} = (8F \times c_{NH_3} \times V)/(M_{NH_3} \times Q) \quad (3)$$

where F is the Faradaic constant (96485 C mol^{-1}) and Q is the total charge during electrolysis.

Isotope labelling experiment

Using $K^{15}NO_3$ (^{15}N-labeled potassium nitrate, Macklin Co., Ltd, with a purity of >99.0%) as the feed nitrogen source, the isotope labeled NITRR was carried out to determine the source of NH_3. 1 M KOH was used as the electrolyte, and 100 mM $K^{15}NO_3$ was added to the cathodic compartment as the reactant. ^1H nuclear magnetic resonance (NMR) spectroscopy was employed to confirm the $^{15}NH_4^+$ and $^{14}NH_4^+$. In a standard procedure, 250 μL of the tested electrolyte was initially diluted to bring it within the detection range and adjusted to a pH of 2.0 by adding 4 M H_2SO_4. Subsequently, the solution was mixed with 250 μL of DMSO-d6 (hexadeuterodimethyl sulfoxide, Shanghai Macklin Biochemical Technology Co., Ltd., with a purity of 99.9%), which included 10 mM $C_4H_4O_4$ (maleic acid, Shanghai Macklin Biochemical Technology Co., Ltd., with a purity of ⩾99.0%). DMSO-d6 served as the solvent, and $C_4H_4O_4$ acted as the internal standard. Finally, the prepared solution was analyzed using a 600 MHz NMR spectrometer.

Assembly of Zn-nitrate batteries and electrochemical test

For the Zn-NO_3^- battery measurement, the Ni foam-supported E-CuBDC (0.5 × 0.5 cm^2) and Zn plate (1.5 × 2 cm^2) were employed as the cathode and anode respectively. Ni foam provides a large specific surface area to support more catalysts and acts as the current collector to speed up the transfer of charge in catalytic reactions. A typical H-type cell that contains 30 mL cathodic electrolyte (1 M KOH + 0.05 M KNO_3) and 30 mL anodic electrolyte (1 M KOH) separated by the Nafion 117 membrane was

assembled. Open circuit voltage (OCV) and the chronoamperometric curves were test using the Neware battery system (CT-4008T-5V50mA-164, Shenzhen, China). Discharging polarization curves with a scan rate of 10 mV s^{-1} were conducted on CHI660E. The power density (P) of Zn-NO_3^- battery was determined as the equation:

$$P = I \times V \quad (4)$$

Where I and V are the discharge current density and voltage, respectively.

In situ ATR-SEIRAS measurements

To detect the reaction intermediates during the NITRR process, the in-situ ATR-SEIRAS spectra were carried out by an FT-IR spectrometer equipped with an MCT-A detector (Nicolet iS50, Thermo Scientific). Firstly, the catalyst inks (10 μL) were dropped onto the central surface of the hemicylindrical Si prism on which an Au film was chemically deposited. The Si prism was assembled in a spectro-electrochemical cell, and the Pt wire was used as the counter electrode, Ag/AgCl electrode was employed as the reference electrode, and 1 M KOH solution with 0.1 M KNO_3 was dispensed as the electrolyte. All spectrum was collected at a resolution of 4 cm^{-1}, and each single-beam spectrum was an average of 200 scans. The electrochemical workstation of Autolab PGSTAT 204 (Metrohm) was used for potential control.

Online differential electrochemical mass spectrometry (DEMS)

The online DEMS measurements were conducted to capture the volatile intermediates and products. For the online DEMS tests, CP coated with E-CuBDC, platinum wire, and Ag/AgCl electrode were utilized as working electrode, counter electrode, and reference electrode, respectively, in a specially designed probe-type DEMS cell. The signal was acquired using a hydrophobic polytetrafluoroethylene (PTFE) membrane. The generated products were transported to the mass spectrometer via a pump system. The NITRR was tested at the applied potential (-0.65 V vs. RHE) and applied alternately, with a reaction interval of 2 min. There is a rest interval between processes before the intermediate signal returns to its initial state. When the electrochemical test concluded

and the mass signal reverted to baseline, the subsequent cycle started under identical conditions to mitigate any potential errors.

COMSOL Multiphysics

The COMSOL Multiphysics simulation of the as-built model was performed by using the different modules. According to the experimental results, the diameter of Cu_2O/Cu nanoparticles (the orange semicircle) was established as 5 nm. In the simulation process, the H_2O molecule and NO_3^- diffused to the surface of catalysts. The "Transport of Diluted Species" module was used to simulate the diffusion of H_2O and NO_3^-. Reaction-related parameters were fitted according to the experimental data, where the transport equation is applied as follows:

$$\frac{\partial c_i}{\partial_t} + \nabla J_i + u \nabla C_i = R_i \qquad (5)$$

$$J_i = -D_i \nabla C_i \qquad (6)$$

Where C_i, t, J_i, u, R_i, and D_i are the concentration of NO_3^- and H_2O, time, diffusion flux, flow rate, source, and diffusion coefficient, respectively.

Molecular dynamics

Molecular dynamics (MD) simulations were carried out by using the Large-scale Atomic/Molecular Massively Parallel Simulator (LAMMPS23) open source software[1]. The simulation system was constructed using the PACKMOL package[2] by stacking a liquid phase onto the CuBDC surface. Based on experimental results and parameters reported in the literature, surface models of the Cu_2O (111) and CuBDC (001) crystal planes were constructed. After adding the solution, the model dimensions were a = 33.0 Å, b = 41.0 Å, and c = 67.0 Å. The NO_3^- was modelled by the cvff force field[3]. The Lennard−Jones potential force field combined with the single point charge (SPC) model for water[4] were used to describe all bonded and nonbonded interactions[5]. The Lennard−Jones interaction parameters between different atom pairs were derived from the Lorentz-Berthelot mixing rules. The O−H bond distance and H−O−H angle in water are kept at 1.0 Å and 109.47° by using the SHAKE algorithm in LAMMPS[6]. The

adsorption models were used with periodic boundary conditions, and the cutoff radius was set to 1 nm. Before the MD simulations, energy minimization was performed using the steepest descent method with a convergence criterion of $1E^{-4}$ kcal/mol. Subsequently, 25 ns MD simulations were conducted. In all MD simulations, atomic motion was described by classical Newton's equations, which were solved using the velocity-Verlet algorithm. A timestep of 0.1 fs was employed. During the MD simulations, the NPT ensemble (300 K, 1 bar) was used, with temperature and pressure controlled by the Nose-Hoover thermostat and Parrinello-Rahman barostat, respectively, to ensure system stability under the given conditions. Additionally, the density distribution of NO_3^- along the z-axis was calculated. All visualization and image rendering were performed using OVITO software[7].

Density functional theory

Density-functional theory (DFT) calculations were carried out using the Cambridge Sequential Total Energy Package (CASTEP),[8] which utilizes the pseudopotential plane wave (PPW) method. The interactions between electrons and ions were described utilizing ultrasoft (USP) potentials. A plane-wave basis set was employed to expand the wave functions, with a cutoff kinetic energy set at 400 eV. For the electron-electron exchange and correlation interactions, the functional parametrized by Perdew-Burke-Ernzerhof (PBE), a form of the general gradient approximation (GGA), was used throughout.[9] The van der Waals interaction was described using the DFT-D2 method proposed by Grimme.[10] During the geometry optimizations, the bottom atoms were fixed at the bulk position. In this work, the Brillouin-zone integrations were conducted using Monkhorst-Pack (MP) grids of special points with the separation of 0.07 $Å^{-1}$ for the model cell. The convergence criterion for the electronic self-consistent field (SCF) loop was set to 1×10^{-6} eV/atom. The atomic structures were optimized until the residual forces were below 0.03 eV $Å^{-1}$. During theoretical calculations, the bottom one layer of Cu (111)/Cu_2O (111) models (see Figure S29) was constrained, and the remaining part was allowed to be relaxed. The Cu (111)/Cu_2O (111) models were loaded onto the surface of CuBDC to form the E-CuBDC model (see Figure S30), as candidates for the

nitrate electroreduction to ammonia. The optimized lattice parameters of E-CuBDC were as follows: a = 13.13 □, b = 12.59 □, c = 27.87 □; α = 90.00°, β = 90.00°, γ = 90.00°. The solvation effect was not taken into consideration to simplify the calculation and balance the expensive computational cost.[11, 12]

The adsorbed $*NO_3$ group formation energy was defined as

$$\Delta E = E(*NO_3) + 0.5 \cdot E(H_2) - E(*) - E(HNO_3) \tag{7}$$

where $E(*NO_3)$, $E(H_2)$, $E(*)$, and $E(HNO_3)$ represent the electronic energies of adsorbed $*NO_3$, H_2, the slab, and the HNO_3 species, respectively. The ΔE can suggest the adsorption capacity of Cu/Cu_2O and E-CuBDC catalysts.[13]

Based on the computational hydrogen electrode (CHE) method,[14] the Gibbs free energy can be calculated by

$$\Delta G = \Delta E + \Delta E_{ZPE} - T \cdot \Delta S + \Delta G_{pH} + \Delta G_{U} \tag{8}$$

where ΔE, ΔE_{ZPE}, and ΔS are the reaction energy, the changes of zero-point energy (ZPE), and entropy (S), respectively. The vibration frequencies were analyzed based on the DFT calculations, and only the adsorbed intermediates were considered for reducing the computational cost. T is the reaction temperature (T= 298.15 K). The ZPE and S could be obtained using harmonic approximation. The free energy contribution of pH (ΔG_{pH}) is based on the H^+ concentration, which could be calculated by

$$\Delta G_{pH} = 2.303 \cdot \kappa_B T \cdot \text{pH} \tag{9}$$

where κ_B is the Boltzmann constant and the pH is set to zero. The ΔG_U term is the contribution of the applied potential (U), which can be computed by $-eU$. To avoid calculating the adsorption free energy of charged NO_3^- (ΔG_{*NO3}), the gaseous HNO_3 was used as a reference. The ΔG_{*NO3} could be calculated using the following expression

$$\Delta G_{*NO3} = G_{*NO3} - G_{*} - G_{HNO3\,(g)} + 1/2 \cdot G_{H2\,(g)} + \Delta G_{correction} \tag{10}$$

where G_{*NO3}, G_{*}, $G_{HNO3\,(g)}$, and $G_{H2\,(g)}$ are the Gibbs free energies of the corresponding species, respectively. The $\Delta G_{correction}$ term is the correction of the dissociation energy from the gaseous HNO_3 to $H^+ + NO_3^-$.[15]

2. Supplementary Figures

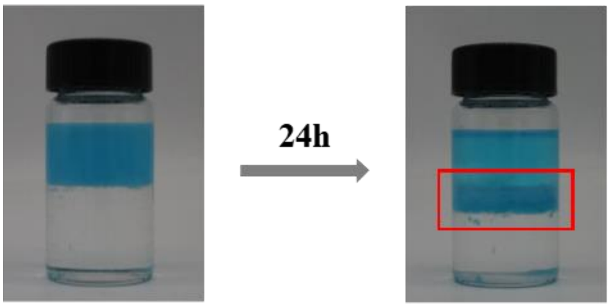

Figure S1 Photograph of the CuBDC products forming between the interface of two solutions.

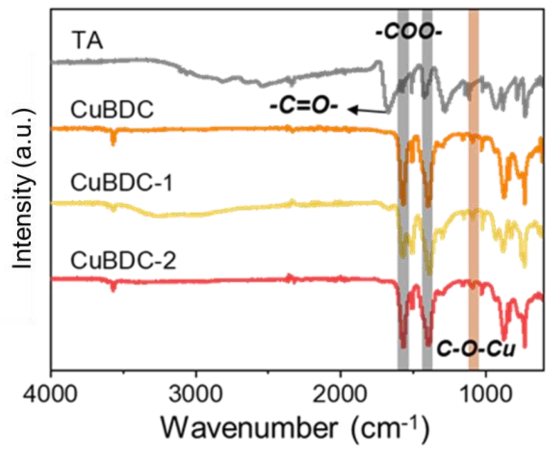

Figure S2 FTIR spectra of TA, CuBDC, CuBDC-1, and CuBDC-2.

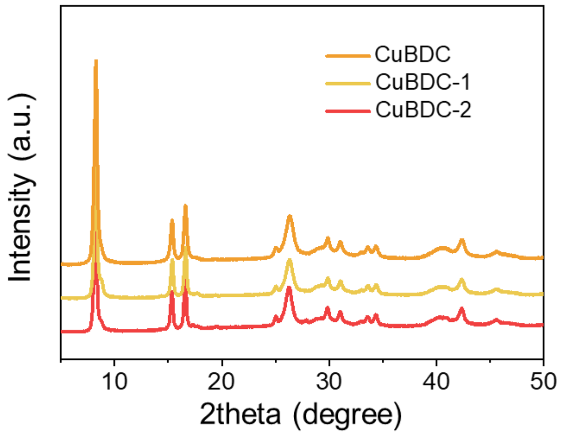

Figure S3 XRD patterns of CuBDC, CuBDC-1, and CuBDC-2.

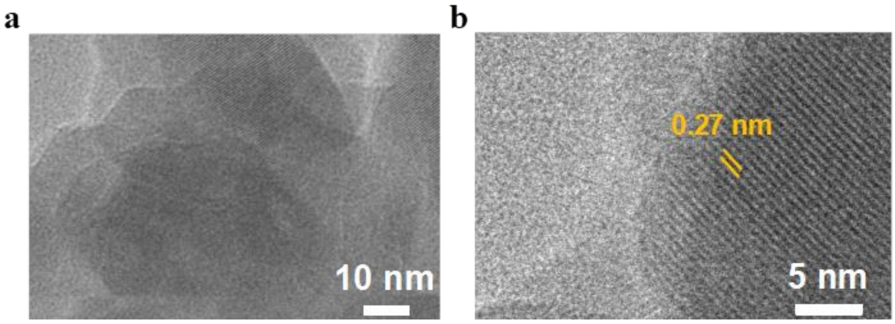

Figure S4 (a, b) TEM images of CuBDC with different magnifications.

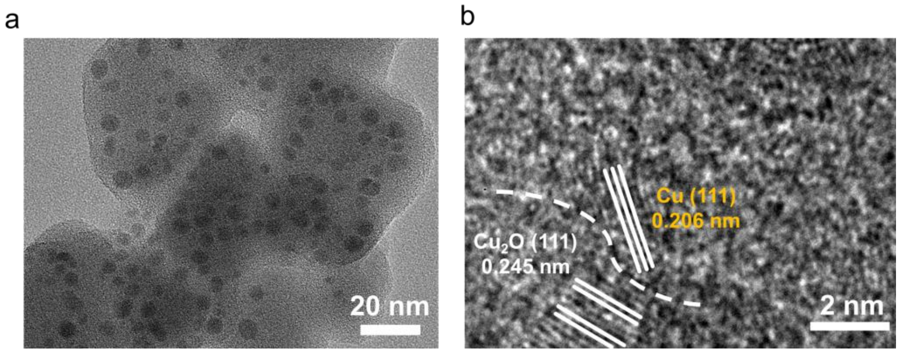

Figure S5 (a, b) Low and high-magnification TEM images of interface of Cu/Cu_2O nanoparticles (NPs) and CuBDC in E-CuBDC.

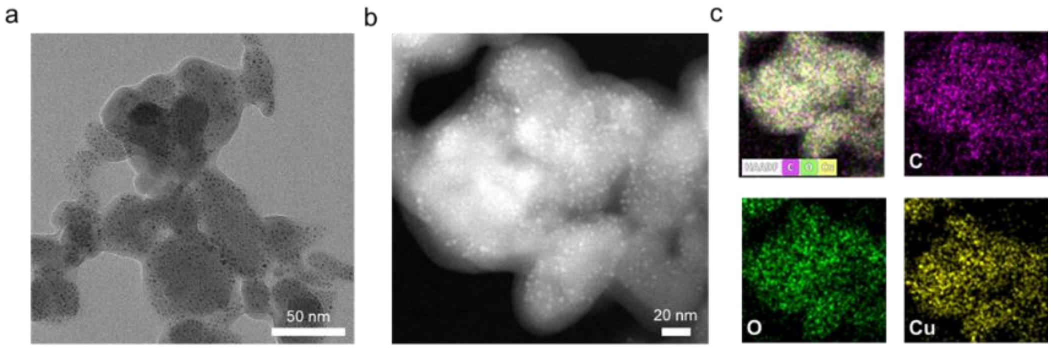

Figure S6 (a) TEM image, (b) high-angle annular dark-field scanning TEM image, and (c) the corresponding element mappings of E-CuBDC.

As illustrated in Figure S6a, the TEM image of E-CuBDC reveals a uniform dispersion of Cu/Cu_2O NPs with a consistent size distribution, confirming their incorporation within the CuBDC support after electrochemical activation. Moreover, the homogeneous distribution of C, O, and Cu elements throughout the E-CuBDC, rather than being confined solely to the Cu/Cu_2O NPs, further reinforces the co-existence of Cu/Cu_2O and CuBDC.

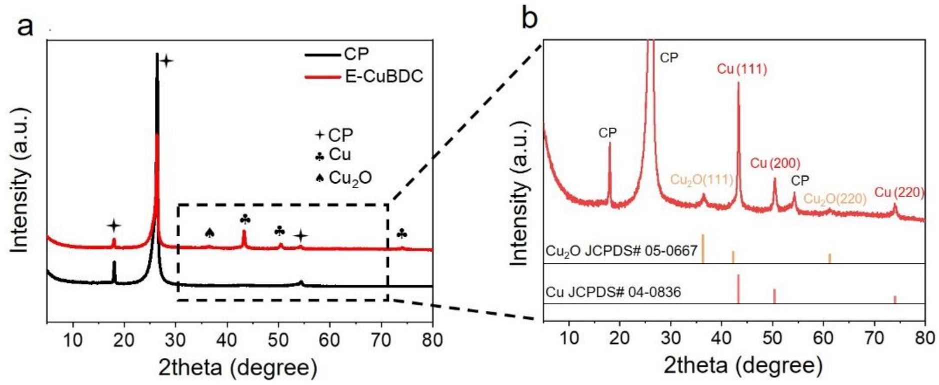

Figure S7 (a) XRD patterns and (b) magnified view of CP and E-CuBDC.

As shown in Figure S7, the diffraction peaks at 36.4°, 43.3°, 50.4°, 61.3°, and 74.1° appear in the XRD patterns of E-CuBDC compared to CP, corresponding to the Cu_2O(111), Cu(111), Cu(200), Cu_2O(220), and Cu (220) planes, respectively. Moreover, the transformation from a crystalline to an amorphous state during the electrochemical activation confirms that the CuBDC underwent structural reconstruction. This illustrates that the Cu/Cu_2O NPs are introduced on the surface of CuBDC during the electrochemical reduction.

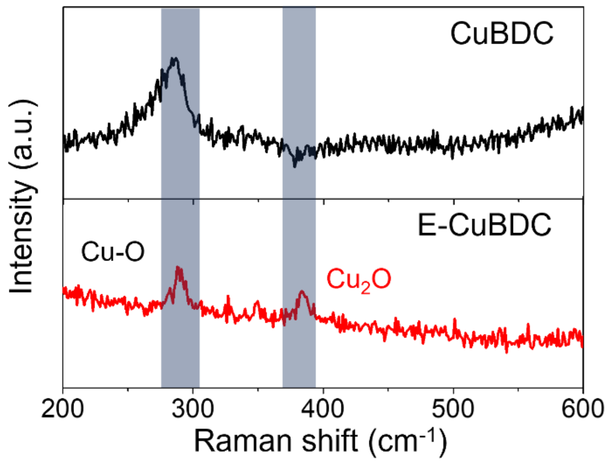

Figure S8 Raman spectra of the CuBDC and E-CuBDC.

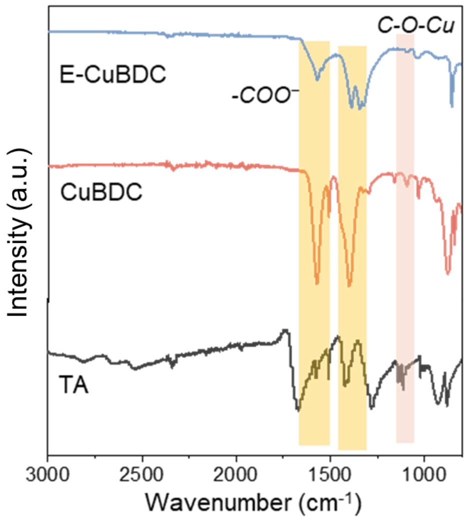

Figure S9 FTIR spectra of TA, CuBDC, and E-CuBDC.

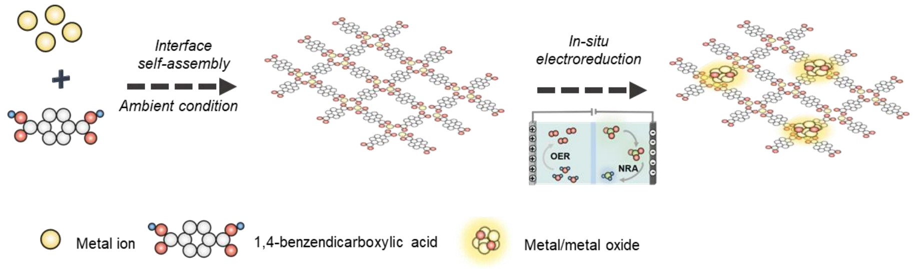

Figure S10 The synthesis and reaction mechanism of the polymer-metal interface in E-CuBDC.

Supporting Note 1:

Based on the experimental results, Figure S10 schematically depicts the evolution of the catalyst and the active species. During the overall electrochemical NITRR process, the evolution of CuBDC consists of two steps. Firstly, at the low reduction potential region, the partial cleavage of coordination bonds between carboxyl groups and Cu^{2+} ions destroyed the original CuBDC. Subsequent cleavage of the coordination bonds exposed Cu^{2+} sites, leading to the structural reconstruction and the formation of CuO NPs. Subsequently, at larger overpotentials, the original Cu(II) species in the formed CuO NPs is partially reduced to Cu_2O metallic Cu(0), forming the Cu/Cu_2O heterostructure NPs on the surface of CuBDC. The reconstructed metal-polymer interface promotes the stability of adaptive Cu /Cu_2O NPs.

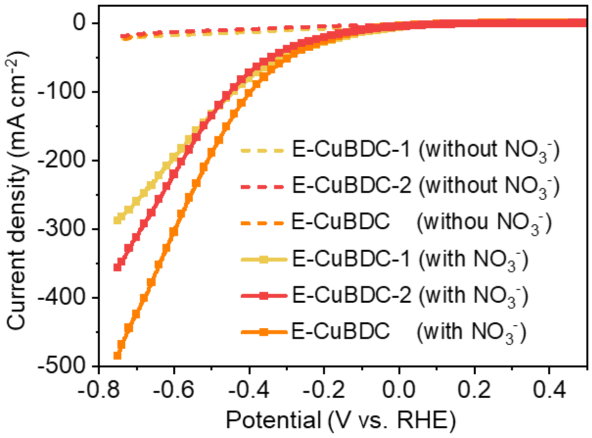

Figure S11 LSV curves of the E-CuBDC-x samples measured in the electrolyte with and without NO_3^-.

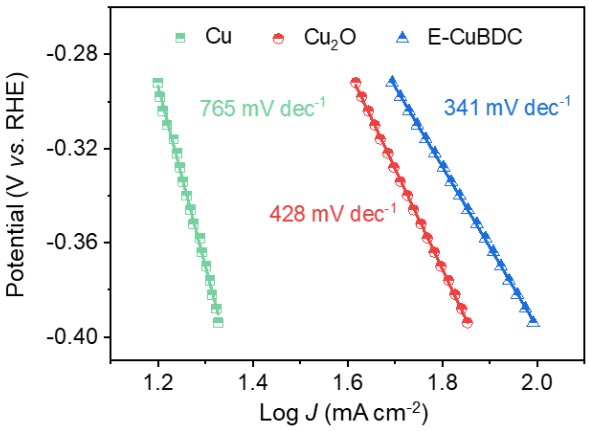

Figure S12 Tafel slopes of the Cu, Cu_2O, and E-CuBDC samples measured in the electrolyte with NO_3^-.

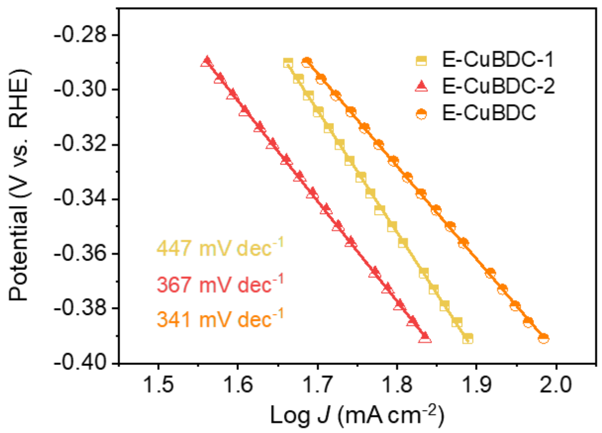

Figure S13 Tafel slopes of the E-CuBDC-x samples measured in the electrolyte with NO_3^-.

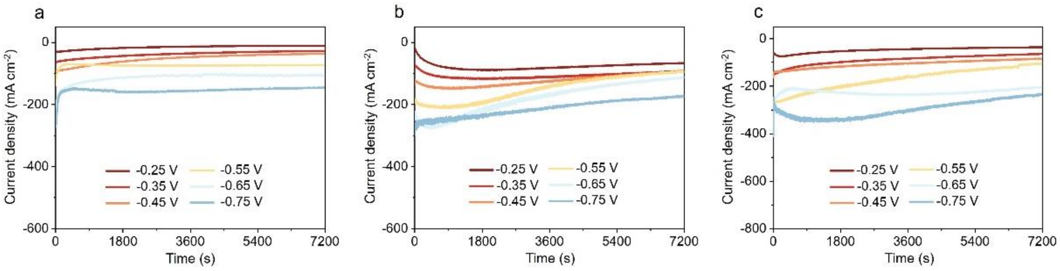

Figure S14 (a-c) The *i–t* curves of Cu, Cu_2O, and E-CuBDC under various applied potentials.

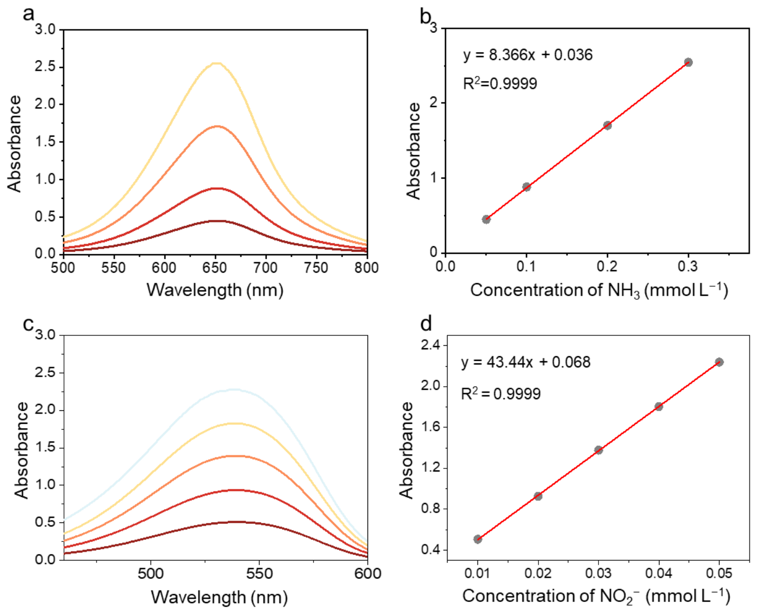

Figure S15 (a, b) The ultraviolet absorption and concentration-absorbance calibration curves of NH_3. (c, d) The ultraviolet absorption and concentration-absorbance calibration curves of NO_2^-.

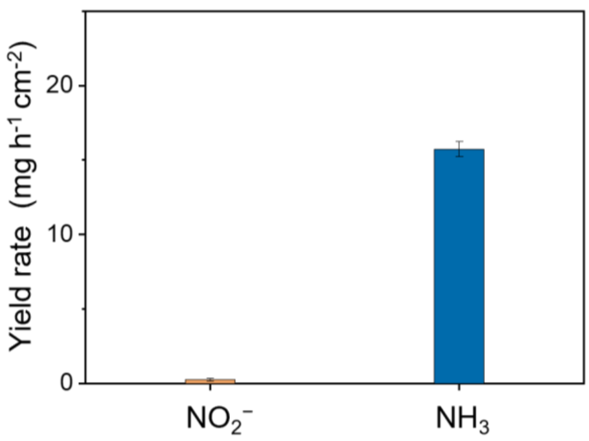

Figure S16 The yield rate of NO_2^- and NH_3 of E-CuBDC at -0.65 V.

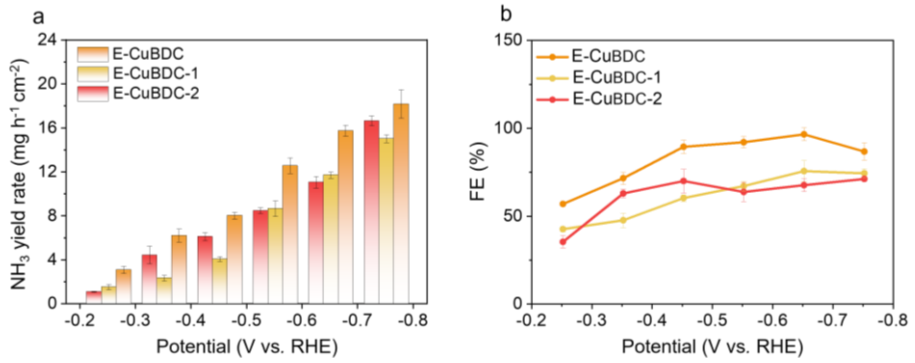

Figure S17 (a) NH_3 yield rate and (b) FE_{NH_3} of the E-CuBDC, E-CuBDC-1, and E-CuBDC-2 samples.

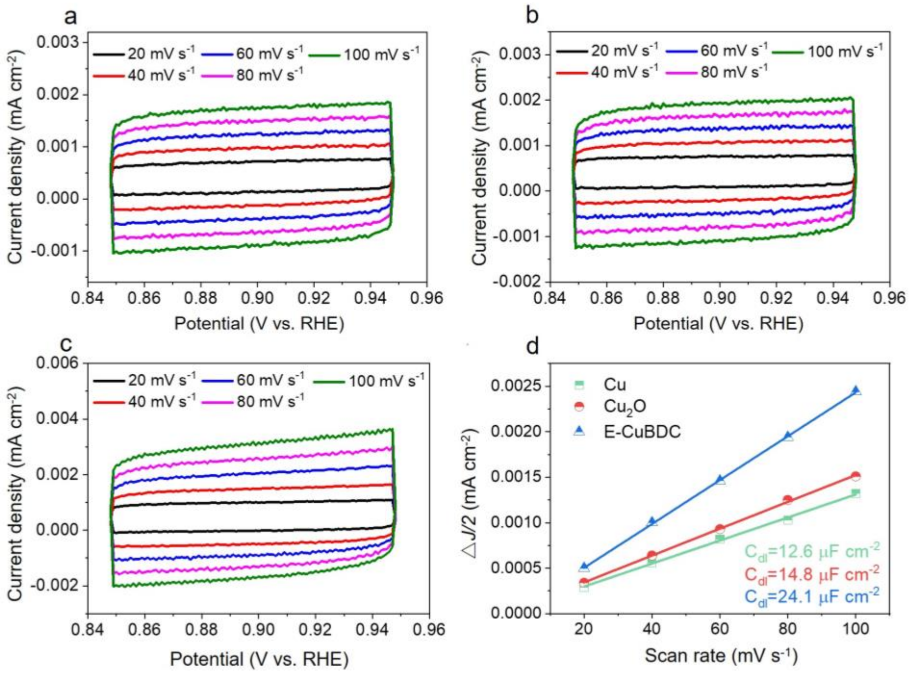

Figure S18 (a-c) CV curves of Cu, Cu_2O, and E-CuBDC catalysts at different scan rates. (d) The measured capacitive currents are plotted as a function of scan rates.

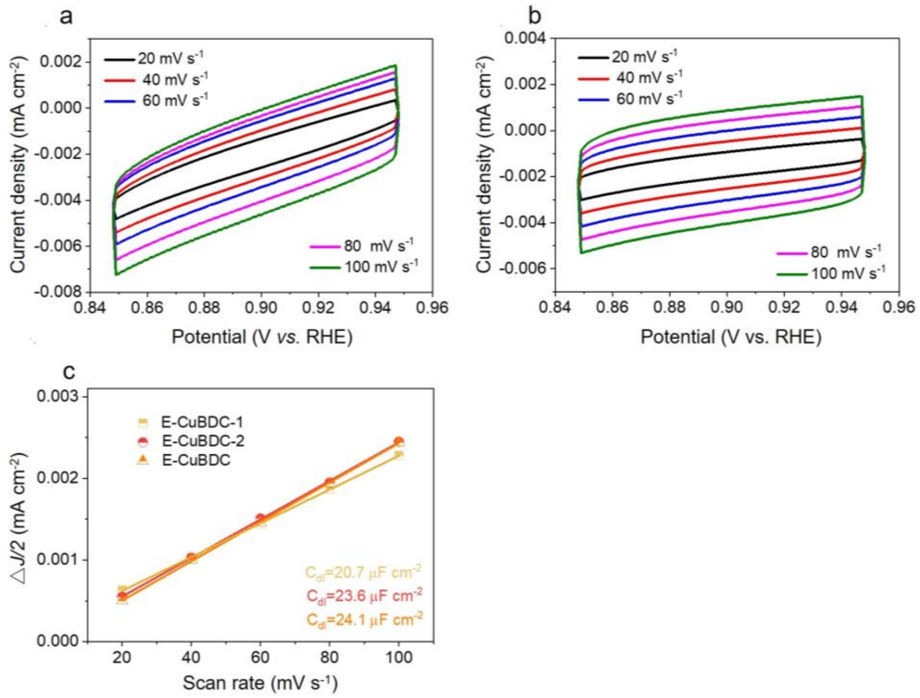

Figure S19 (a, b) CV curves of E-CuBDC-1 and E-CuBDC-2 in the potential window of 0.848-0.948 V (vs. RHE) at different scan rates. (c) The corresponding measured capacitive currents are plotted as a function of scan rates.

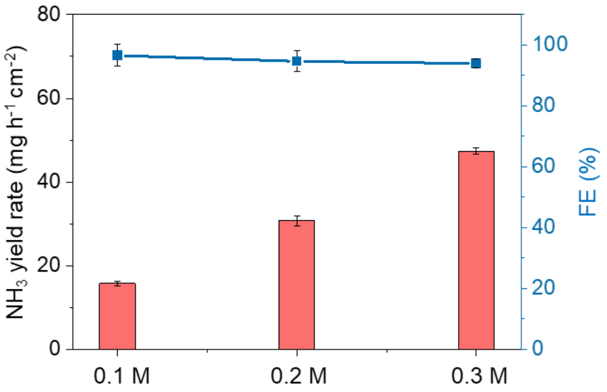

Figure S20 FE_{NH_3} and NH_3 yield rate of E-CuBDC with various NO_3^--N concentration from 0.1 M to 0.3 M.

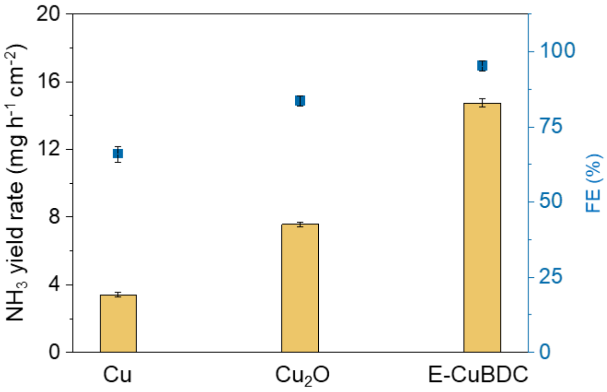

Figure S21 FE_{NH_3} and NH_3 yield rate of Cu, Cu_2O, and E-CuBDC under neutral conditions at the optimal potential.

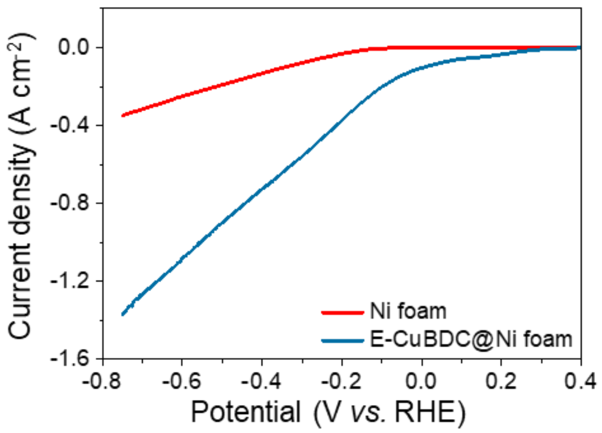

Figure S22 LSV curves of Ni foam and CuBDC@Ni foam in 1 M KOH with 0.1 M NO_3^--N.

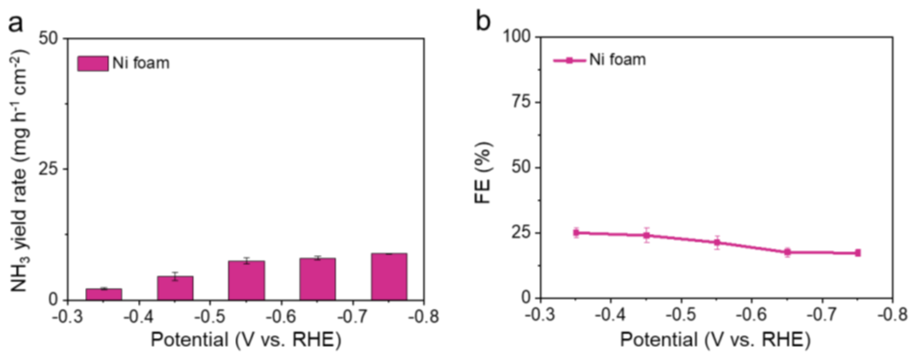

Figure S23 (a) NH_3 yield rates and (b) FE_{NH_3} of the Ni foam substrate.

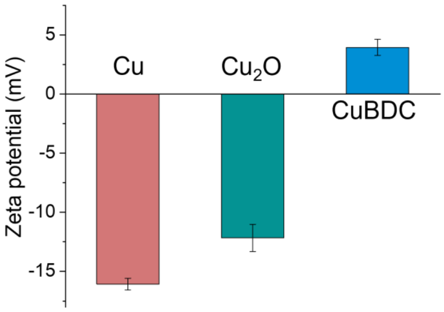

Figure S24 The surface Zeta potential of Cu, Cu_2O, and CuBDC.

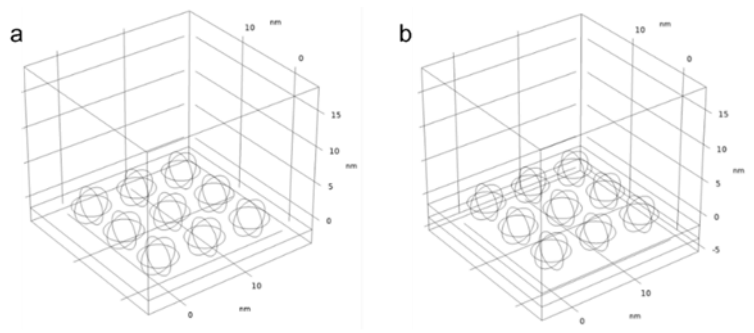

Figure S25 Simulation domains of (a) Cu/Cu_2O and (b) E-CuBDC in the Finite-element simulations.

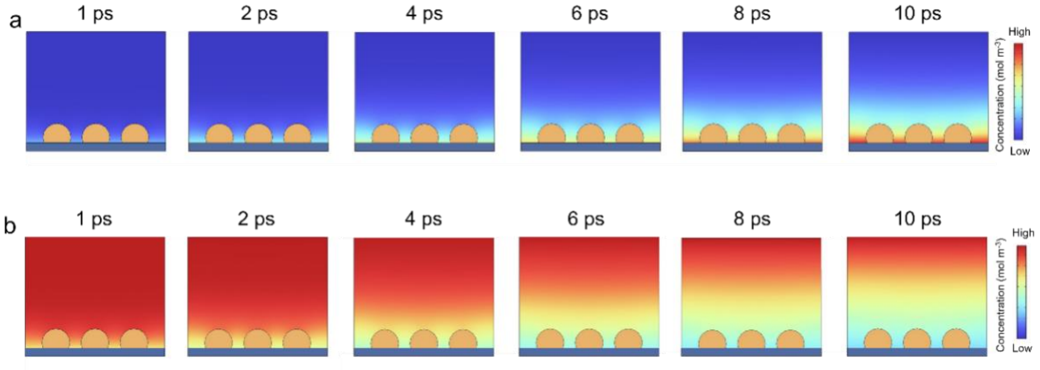

Figure S26 The concentrations evolution of (a) NO_3^- and (b) H_2O on the surface of E-CuBDC with different times.

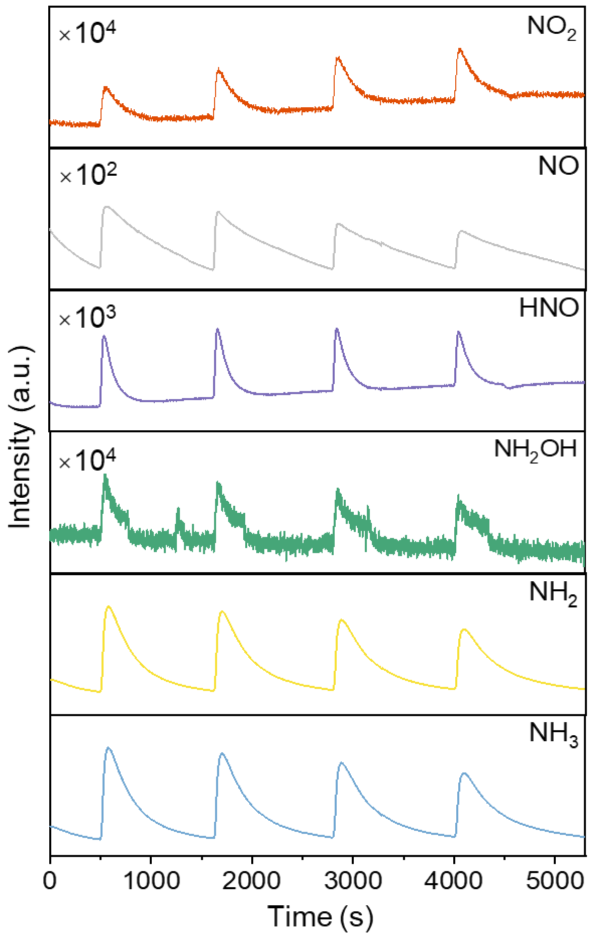

Figure S27 DEMS measurements under NITRR operation for E-CuBDC.

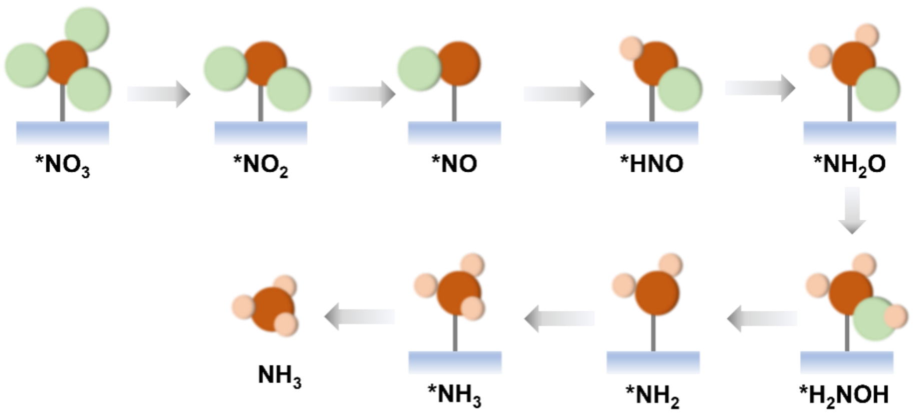

Figure S28 Schematic illustration for the NITRR reaction pathway over E-CuBDC.

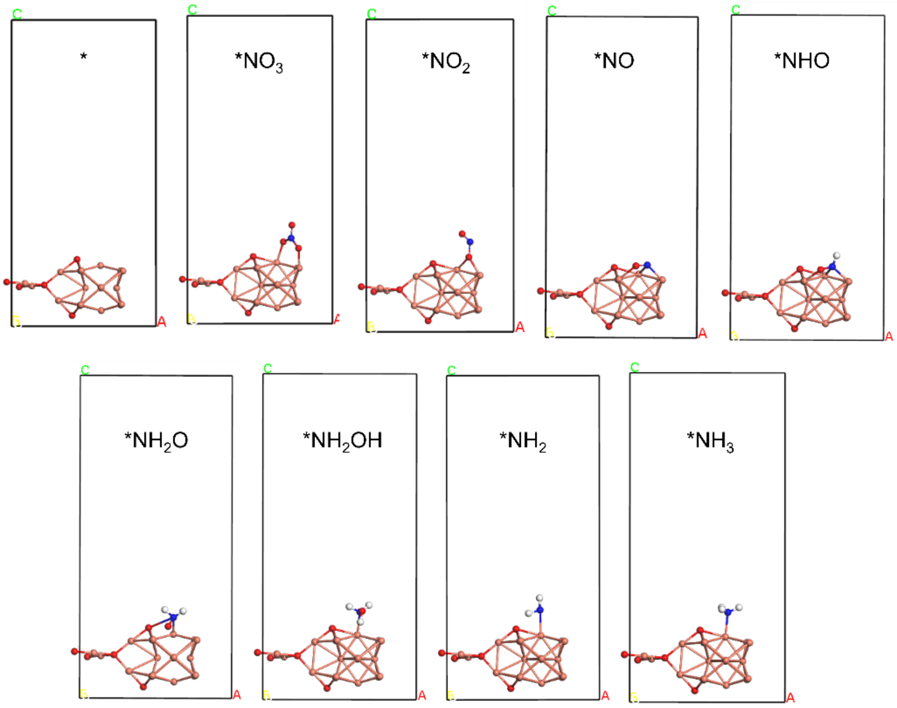

Figure S29 The optimized Cu/Cu_2O structures with different intermediates during the electrocatalytic NITRR processes.

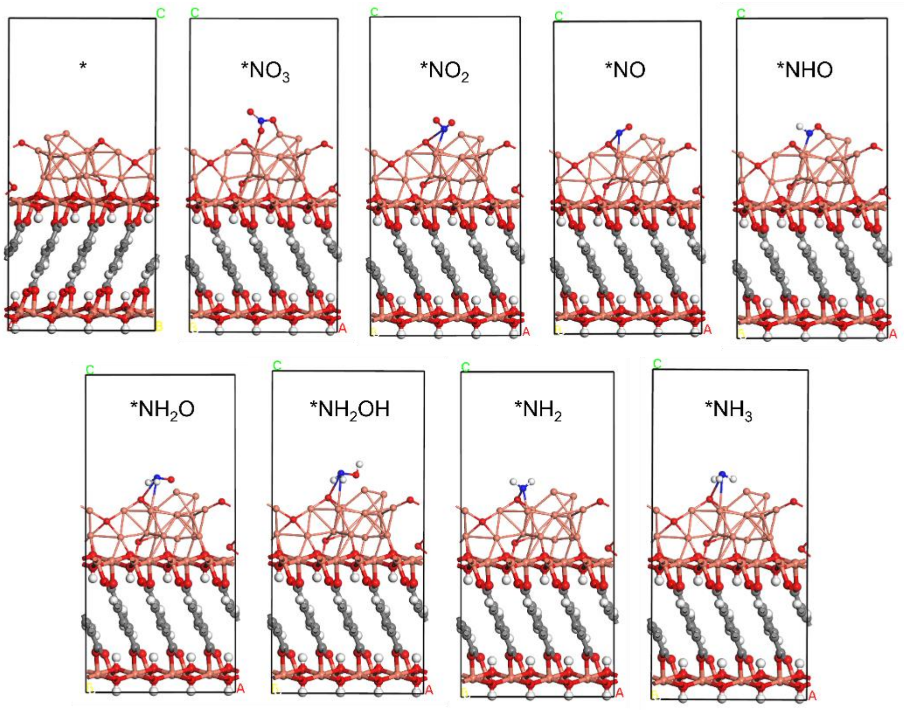

Figure S30 The optimized E-CuBDC structures with different intermediates during the electrocatalytic NITRR process.

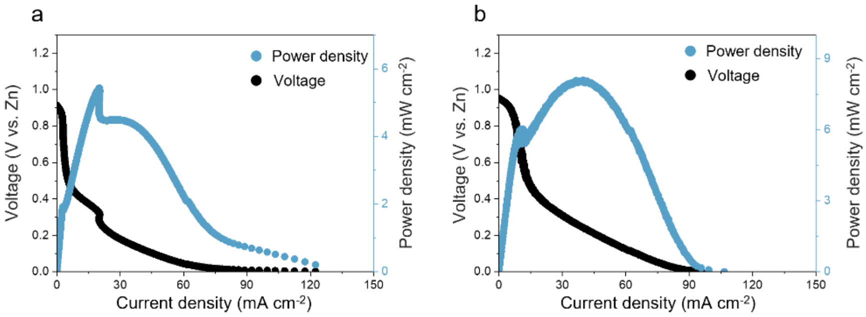

Figure S31 Discharge curves and power densities of Zn-NO_3^- battery using (a) Cu and (b) Cu_2O cathode.

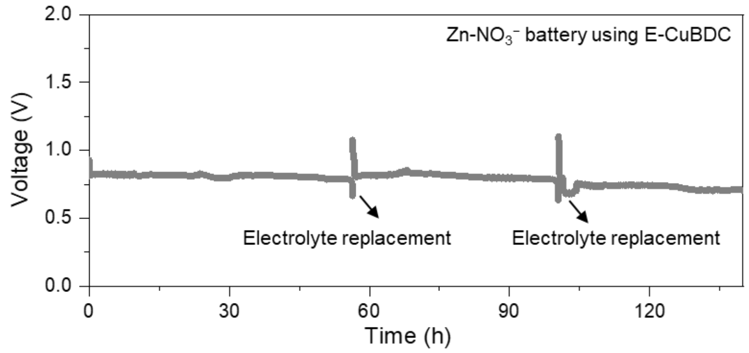

Figure S32 The long-term stability of $Zn-NO_3^-$ battery using E-CuBDC cathode.

3. Supplementary Tables

Table S1. Performance comparison for E-CuBDC, Cu_2O, and Cu catalysts measured under various NO_3^--N concentrations.

Material	NO_3^--N concentration (mg L^{-1})	FE (%)	NH_3 yield rate (mg h^{-1} cm^{-2})
Cu	25	13.2	0.04
	50	13.4	0.12
	100	44.6	0.39
	200	48.2	0.55
	500	47.2	1.91
	1000	51.5	2.25
Cu_2O	25	23.0	0.14
	50	33.4	0.53
	100	57.5	0.75
	200	60.2	1.25
	500	60.3	2.76
	1000	60.1	3.81
E-CuBDC	25	53.1	0.67
	50	78.7	2.04
	100	91.3	3.19
	200	91.5	4.64
	500	91.4	5.89
	1000	93.2	10.47

Table S2 Comparing the NH_3 yield rates and FEs of E-CuBDC and other reported catalysts towards NITRR.

**Cathode**	**Electrolyte**	**FE (%)**	**NH_3 Yield rate**	**Ref.**
Cu-NBs-100	1 M KOH + 0.1 M KNO_3	95.3	650 mmol g_{cat}^{-1} h^{-1}	16.
$CuCo_2O_4$/CFs	1 M KOH + 0.1 M NO_3^-	81.9	394.5 mmol h^{-1} g^{-1}<br>0.1578 mmol h^{-1} cm^{-2}	17.
$Cu_{50}Co_{50}$	1 M KOH + 100 mM NO_3^-	100	4.83 mmol h^{-1} cm^{-2}<br>960 mmol g_{cat}^{-1} h^{-1}	18.
Co-Fe@Fe_2O_3	0.1 M Na_2SO_4 + 500 ppm $NaNO_3$	85.2	1505.9 µg h^{-1} cm^{-2}	19.
Cu@C	1 M KOH + 1 mM KNO_3	72.0	469.5 µg h^{-1} cm^{-2}	20.
Fe/Cu-NG	1 M KOH + 0.1 M KNO_3	92.5	1.08 mmol h^{-1} mg^{-1}	21.
Ru_1Cu_{10}/rGO	1 M KOH + 0.1 M KNO_3	98	0.38 mmol cm^{-2} h^{-1}	22.
$Cu_{50}Ni_{50}$	0.01 M KOH + 0.5 M Na_2SO_4 + 100 mM NO_3^-	88.0	583.6 µmol cm^{-2} h^{-1}	23.
PR-CuNC	0.1 M KOH + 0.1 M KNO_3	94.61	3.74 mg h^{-1} cm^{-2}	24.
PTCDA/O-Cu	0.1 M PBS + 500 ppm NO_3^-	77	436 µg h^{-1} cm^{-2}	25.
Cu-N-C SAC	1.0 M KOH + 0.1 M KNO_3	84.7	12.5 mol g_{Cu}^{-1} h^{-1}	26.

CuCoSP	0.1 M KOH + 0.1 M KNO_3	93.3	1.17 mmol h^{-1} cm^{-2}	27.
Cu/Cu_2O	0.5 M Na_2SO_4 + 200 ppm NO_3^--N	95.8	0.2449 mmol h^{-1} cm^{-2}	28.
Cu nanodisks (NDs)	0.1 M KOH + 10 mM KNO_3	81.1	2.16 mg h−1 g_{cat}^{-1}	29.
Cu-N-C	0.5 M Na_2SO_4 + 50 mg L^{-1} $NaNO_3$-N	94.0	9.23 mg h^{-1} g_{cat}^{-1}	30.
Rh@Cu-0.6%	0.1 Na_2SO_4 + 0.1 M KOH	93	1.27 mmol h^{-1} cm^{-2}	31.
PA-RhCu cNCs	0.1 M $HClO_4$ + 0.05 M KNO_3	93.7	2.40 mg h^{-1} g_{cat}^{-1}	32.
Ru-CuNW	1 M KOH + 2000 ppm NO_3^-	96	76,500 µg h^{-1} cm^{-2}	33.
Cu-O_v-W	0.5 M Na_2SO_4 + 0.05 M NO_3^-	94.6	5.84 mg h^{-1} g_{cat}^{-1}	34.
V_{Cu}-Au_1Cu SAAs	0.1 M KOH + 7.14 mM KNO_3	98.7	0.555 mg h^{-1} cm^{-2}	35.
Cu-SnS_{2-x}	0.1 M KOH+ 0.1 M KNO_3	93.8	0.63 mmol h^{-1} g_{cat}^{-1}	36.
V-Cu NAE	0.5M K_2SO_4 + 200 ppm nitrate-N	95.1	7.83 mg h^{-1} cm^{-2}	37.
Cu/Fe 2D MOF	0.1M Na_2SO_4 + 200 mg L^{-1} NO_3-N	62.8	0.2268 mg h^{-1} cm^{-2} g_{cat}^{-1}	38.
Cu-BTC-Cu	0.1M Na_2SO_4 + 750 mg L^{-1} KNO_3-N	83.8	4.00 mg h^{-1} cm^{-2} g_{cat}^{-1}	39.
E-CuBDC	1 M KOH +0.1 M KNO_3	96.6	15.7 mg h^{-1} cm^{-2}	This work

Table S3 Performance comparisons of the Zn-NO_3^- battery with E-CuBDC cathode and other reported Zn-nitrogen batteries.

Batteries	Anolyte	Catholyte	Cathode	Stability (h)	Power density (mW cm^{-2})	NH_3 yield (mg h^{-1} cm^{-2})	FE (%)	Ref.
Zn-NO_3^-	1 M KOH	1 M KOH + 0.05 M KNO_3	E-CuBDC	145	17.9	3.44	82.2	This work
Zn-NO_3^-	1 M KOH	1 M KOH + 0.1 M $NaNO_3$	0.6W-O-CoP	N/A	9.27	2.79	75.6	40.
Zn-NO_3^-	6 M KOH	0.1 M $HClO_4$ + 0.05 M KNO_3	RhNi@Rh BMLs	N/A	10.5	2.66	92.7	41.
Zn-NO_3^-	6 M KOH + 0.2 M $Zn(Ac)_2$	0.5 M K_2SO_4 + 0.05 M KNO_3	CuTABQ	~18.3	12.3	--	98.4	42.
Zn-NO_3^-	1 M KOH	0.5 M NO_3 + 1 M KOH	NiRu	~80	10.0	--	--	43.
Zn-NO_3^-	1 M KOH	0.5 M Na_2SO_4 + 0.1 M $NaNO_3$	ISAA In-Pdene	70	12.64	3.199	93.4	44.
Zn-NO_3^-	1 M KOH	0.5 M Na_2SO_4 +100 ppm NO_3^--N	Co–B@CoO_x	8	4.78	0.89	85.4	45.
Zn-NO_3^-	1 M KOH	1 M KOH + 0.5 M KNO_3	2D Cu plates	N/A	12.09	0.89	85.4	46.
Zn-NO_3^-	1 M KOH + 0.02 M $Zn(Ac)_2$	0.5 M Na_2SO_4 + 3000 ppm NO_3^- + 1 M EtOH	RhCu M-tpp	~43	1.54		76.9	47.
Zn-NO_3^-	5 M KOH	0.25 M $LiNO_3$ + 5	Pd/TiO_2	12	0.87	0.54	81.3	48.

		M LiCl						
Zn-NO_3^-	1 M KOH	0.2 M K_2SO_4 + 0.05 M KNO_3	Fe/Ni_2P	~3	3.25	0.38	85	49.
Zn-NO_3^-	1 M KOH	0.5 M Na_2SO_4 + 0.1 M KNO_3	Bi_1-$CuCo_2O_4$	~3	2.81	0.62	97.76	50.
Zn-NO_3^-	1 m KOH	1 m KOH + 0.1 m KNO_3	Cu_9S_5	~0.33	12.27	1.49	84.4	51.
Zn-NO_3^-	3 M KOH	6 M KOH + 1 M KNO_3	Ni-MOF-Ru	37	4.99	2.1	85.3	52.
Zn-NO_3^-	1 M KOH	1 M KOH + 0.1 M $NaNO_3$	Sb/Bi	~6	11.88	1.48	Zn-NO_3^-	53.
Zn-NO_2^-	1 M KOH	0.5 M K_2SO_4 + 0.05 M KNO_2	C/Co_3O_4	24	6.03	0.8	95.1	54.
Zn-NO_2^-	1 M NaOH	1 M NaOH + 0.5 M $NaNO_2$	Cu_3P-Fe_2P	N/A	4.34	1.03	96.5	55.
Zn-NO_2^-	1 M KOH	0.5 M K_2SO_4 + 0.05 M KNO_2	Cu_xNi_y/MXene	24	8.34	--	90.3	56.
Zn-NO	1 M KOH	0.1 M HCl + 0.5 mM Fe(II)SB	MoS_2 nanosheets	N/A	1.04	--	--	57.
Zn-N_2	1.0 M KOH	1.0 M KOH	CoPi/NPCS	~80	0.49	--	16.35	58.
Zn-N_2	0.1 M KOH	0.1 M KOH	VN@NSC-900	2	0.01642	0.000172	~13	59.
Zn-N_2	0.1 M KOH	0.1 M KOH	$Fe_{1.0}$HTNs	2	0.02765	0.000137	~21	60.

References

[1] Plimpton, S. Fast parallel algorithms for short-range molecular dynamics. *J. Comput. Phys.* **1995**, *117* (1), 1-19.

[2] Martínez, L.; Andrade, R.; Birgin, E. G.; Martínez, J. M. PACKMOL: A package for building initial configurations for molecular dynamics simulations. *J. Comput. Chem.* **2009**, *30* (13), 2157-2164.

[3] Dauber□Osguthorpe, P.; Roberts, V. A.; Osguthorpe, D. J.; Wolff, J.; Genest, M.; Hagler, A. T. Structure and energetics of ligand binding to proteins: Escherichia coli dihydrofolate reductase□trimethoprim, a drug□receptor system. *Proteins: Struct. Funct. Bioinforma.* **1988**, *4* (1), 31-47.

[4] Berendsen, H.-J. C.; Grigera, J. R.; Straatsma, T. P. The missing term in effective pair potentials. *J. Phys. Chem.* **1987**, *91* (24), 6269-6271.

[5] Lorentz, H. A. Ueber die Anwendung des Satzes vom Virial in der kinetischen Theorie der Gase. *Ann. Phys.* **1881**, *248* (1), 127-136.

[6] Ryckaert, J.-P.; Ciccotti, G.; Berendsen, H. J. Numerical integration of the cartesian equations of motion of a system with constraints: molecular dynamics of n-alkanes. *J. Comput. Phys.* **1977**, *23* (3), 327-341.

[7] Stukowski, A. Visualization and analysis of atomistic simulation data with OVITO–the Open Visualization Tool. *Model. Simul. Mat. Sci. Eng.* **2009**, *18* (1), 015012.

[8] Segall, M. D.; Lindan, P. J. D.; Probert, M. J. a.; Pickard, C. J.; Hasnip, P. J.; Clark, S. J.; Payne, M. C. First-principles simulation: ideas, illustrations and the CASTEP code. *J. Phys. Condens. Matter* **2002**, *14* (11), 2717.

[9] Perdew, J. P.; Burke, K.; Ernzerhof, M. Generalized gradient approximation made simple. *Phys. Rev. Lett.* **1996**, *77* (18), 3865.

[10] Grimme, S.; Antony, J.; Ehrlich, S.; Krieg, H. A consistent and accurate ab initio parametrization of density functional dispersion correction (DFT-D) for the 94 elements H-Pu. *J. Chem. Phys.* **2010**, *132* (15).

[11] Guo, X.; Gu, J.; Lin, S.; Zhang, S.; Chen, Z.; Huang, S. Tackling the activity and selectivity challenges of electrocatalysts toward the nitrogen reduction reaction via atomically dispersed biatom catalysts. *J. Am. Chem. Soc.* **2020**, *142* (12), 5709-5721.

[12] Katsounaros, I.; Figueiredo, M. C.; Chen, X.; Calle-Vallejo, F.; Koper, M. T. Structure-and coverage-sensitive mechanism of NO reduction on platinum electrodes. *ACS Catal.* **2017**, *7* (7), 4660-4667.

[13] Chun, H. J.; Apaja, V.; Clayborne, A.; Honkala, K.; Greeley, J. Atomistic insights into nitrogen-cycle electrochemistry: a combined DFT and kinetic Monte Carlo analysis of NO electrochemical reduction on Pt (100). *ACS Catal.* **2017**, *7* (6), 3869-3882.

[14] Skulason, E.; Bligaard, T.; Gudmundsdóttir, S.; Studt, F.; Rossmeisl, J.; Abild-Pedersen, F.; Vegge, T.; Jónsson, H.; Nørskov, J. K. A theoretical evaluation of possible transition metal electro-catalysts for N_2 reduction. *Phys. Chem. Chem. Phys.* **2012**, *14* (3), 1235-1245.

[15] Sathishkumar, N.; Chen, H.-T. Mechanistic understanding of the electrocatalytic nitrate reduction activity of double-atom catalysts. *J. Phys. Chem. C.* **2023**, *127* (2), 994-1005.

[16] Hu, Q.; Qin, Y.; Wang, X.; Wang, Z.; Huang, X.; Zheng, H.; Gao, K.; Yang, H.; Zhang, P.; Shao, M. Reaction intermediate-mediated electrocatalyst synthesis favors specified facet and

defect exposure for efficient nitrate–ammonia conversion. *Energy Environ. Sci.* **2021**, *14* (9), 4989-4997.
[17] Niu, Z.; Fan, S.; Li, X.; Wang, P.; Liu, Z.; Wang, J.; Bai, C.; Zhang, D. Bifunctional copper-cobalt spinel electrocatalysts for efficient tandem-like nitrate reduction to ammonia. *Chem. Eng. J.* **2022**, *450*, 138343.
[18] Fang, J.-Y.; Zheng, Q.-Z.; Lou, Y.-Y.; Zhao, K.-M.; Hu, S.-N.; Li, G.; Akdim, O.; Huang, X.-Y.; Sun, S.-G. Ampere-level current density ammonia electrochemical synthesis using CuCo nanosheets simulating nitrite reductase bifunctional nature. *Nat. Commun.* **2022**, *13* (1), 7899.
[19] Zhang, S.; Li, M.; Li, J.; Song, Q.; Liu, X. High-ammonia selective metal–organic framework–derived Co-doped Fe/Fe_2O_3 catalysts for electrochemical nitrate reduction. *Proc. Natl. Acad. Sci. U.S.A.* **2022**, *119* (6), e2115504119.
[20] Song, Z.; Liu, Y.; Zhong, Y.; Guo, Q.; Zeng, J.; Geng, Z. Efficient electroreduction of nitrate into ammonia at ultralow concentrations via an enrichment effect. *Adv. Mater.* **2022**, *34* (36), 2204306.
[21] Zhang, S.; Wu, J.; Zheng, M.; Jin, X.; Shen, Z.; Li, Z.; Wang, Y.; Wang, Q.; Wang, X.; Wei, H. Fe/Cu diatomic catalysts for electrochemical nitrate reduction to ammonia. *Nat. Commun.* **2023**, *14* (1), 3634.
[22] Gao, W.; Xie, K.; Xie, J.; Wang, X.; Zhang, H.; Chen, S.; Wang, H.; Li, Z.; Li, C. Alloying of Cu with Ru enabling the relay catalysis for reduction of nitrate to ammonia. *Adv. Mater.* **2023**, *35* (19), 2202952.
[23] Bu, Y.; Wang, C.; Zhang, W.; Yang, X.; Ding, J.; Gao, G. Electrical pulse□driven periodic self□repair of Cu□Ni tandem catalyst for efficient ammonia synthesis from nitrate. *Angew. Chem. Int. Ed.* **2023**, *62* (24), e202217337.
[24] Liu, Y.; Qiu, W.; Wang, P.; Li, R.; Liu, K.; Omer, K. M.; Jin, Z.; Li, P. Pyridine-N-rich Cu single-atom catalyst boosts nitrate electroreduction to ammonia. *Appl. Catal. B Environ. Energy* **2024**, *340*, 123228.
[25] Chen, G.-F.; Yuan, Y.; Jiang, H.; Ren, S.-Y.; Ding, L.-X.; Ma, L.; Wu, T.; Lu, J.; Wang, H. Electrochemical reduction of nitrate to ammonia via direct eight-electron transfer using a copper–molecular solid catalyst. *Nat. Energy* **2020**, *5* (8), 605-613.
[26] Yang, J.; Qi, H.; Li, A.; Liu, X.; Yang, X.; Zhang, S.; Zhao, Q.; Jiang, Q.; Su, Y.; Zhang, L. Potential-driven restructuring of Cu single atoms to nanoparticles for boosting the electrochemical reduction of nitrate to ammonia. *J. Am. Chem. Soc.* **2022**, *144* (27), 12062-12071.
[27] He, W.; Zhang, J.; Dieckhöfer, S.; Varhade, S.; Brix, A. C.; Lielpetere, A.; Seisel, S.; Junqueira, J. R. C.; Schuhmann, W. Splicing the active phases of copper/cobalt-based catalysts achieves high-rate tandem electroreduction of nitrate to ammonia. *Nat. Commun.* **2022**, *13* (1), 1129.
[28] Wang, Y.; Zhou, W.; Jia, R.; Yu, Y.; Zhang, B. Unveiling the activity origin of a copper□based electrocatalyst for selective nitrate reduction to ammonia. *Angew. Chem. Int. Ed.* **2020**, *59* (13), 5350-5354.
[29] Wu, K.; Sun, C.; Wang, Z.; Song, Q.; Bai, X.; Yu, X.; Li, Q.; Wang, Z.; Zhang, H.; Zhang, J. Surface reconstruction on uniform Cu nanodisks boosted electrochemical nitrate reduction to ammonia. *ACS Mater. Lett.* **2022**, *4* (4), 650-656.

[30] Chen, H.; Zhang, C.; Sheng, L.; Wang, M.; Fu, W.; Gao, S.; Zhang, Z.; Chen, S.; Si, R.; Wang, L. Copper single-atom catalyst as a high-performance electrocatalyst for nitrate-ammonium conversion. *J. Hazard. Mater.* **2022**, *434*, 128892.
[31] Liu, H.; Lang, X.; Zhu, C.; Timoshenko, J.; Rüscher, M.; Bai, L.; Guijarro, N.; Yin, H.; Peng, Y.; Li, J. Efficient electrochemical nitrate reduction to ammonia with copper□supported rhodium cluster and single□atom catalysts. *Angew. Chem. Int. Ed.* **2022**, *61* (23), e202202556.
[32] Ge, Z. X.; Wang, T. J.; Ding, Y.; Yin, S. B.; Li, F. M.; Chen, P.; Chen, Y. Interfacial engineering enhances the electroactivity of frame□like concave RhCu bimetallic nanocubes for nitrate reduction. *Adv. Energy Mater.* **2022**, *12* (15), 2103916.
[33] Chen, F.-Y.; Wu, Z.-Y.; Gupta, S.; Rivera, D. J.; Lambeets, S. V.; Pecaut, S.; Kim, J. Y. T.; Zhu, P.; Finfrock, Y. Z.; Meira, D. M. Efficient conversion of low-concentration nitrate sources into ammonia on a Ru-dispersed Cu nanowire electrocatalyst. *Nat. nanotechnol.* **2022**, *17* (7), 759-767.
[34] Chen, D.; Zhang, S.; Bu, X.; Zhang, R.; Quan, Q.; Lai, Z.; Wang, W.; Meng, Y.; Yin, D.; Yip, S. Synergistic modulation of local environment for electrochemical nitrate reduction via asymmetric vacancies and adjacent ion clusters. *Nano Energy* **2022**, *98*, 107338.
[35] Zhang, Y.; Chen, X.; Wang, W.; Yin, L.; Crittenden, J. C. Electrocatalytic nitrate reduction to ammonia on defective Au1Cu (111) single-atom alloys. *Appl. Catal. B Environ. Energy* **2022**, *310*, 121346.
[36] Li, H.; Xu, X.; Lin, X.; Chen, S.; He, M.; Peng, F.; Gao, F. Cooperative interaction between Cu and sulfur vacancies in SnS2 nanoflowers for highly efficient nitrate electroreduction to ammonia. *J. Mater. Chem. A* **2023**, *11* (4), 2014-2022.
[37] Zhang, B.; Dai, Z.; Chen, Y.; Cheng, M.; Zhang, H.; Feng, P.; Ke, B.; Zhang, Y.; Zhang, G. Defect-induced triple synergistic modulation in copper for superior electrochemical ammonia production across broad nitrate concentrations. *Nat. Commun.* **2024**, *15* (1), 2816.
[38] Wang, Y.; Cao, Y.; Hai, Y.; Wang, X.; Su, S.; Ding, W.; Liu, Z.; Li, X.; Luo, M. Metal–organic framework-derived Cu nanoparticle binder-free monolithic electrodes with multiple support structures for electrocatalytic nitrate reduction to ammonia. *Dalton Trans.* **2023**, *52* (32), 11213-11221.
[39] Ma, Q.; Xue, Y.; Zhang, C.; Chen, Y.; Teng, W.; Zhang, H.; Fan, J. 2D copper-iron bimetallic metal-organic frameworks for reduction of nitrate with boosted efficiency and ammonia selectivity. *J. Environ. Sci.* **2025**, *149*, 374-385.
[40] Chang, Z.; Meng, G.; Chen, Y.; Chen, C.; Han, S.; Wu, P.; Zhu, L.; Tian, H.; Kong, F.; Wang, M. Dual□site W□O□Cop catalysts for active and selective nitrate conversion to ammonia in a broad concentration window. *Adv. Mater.* **2023**, *35* (32), 2304508.
[41] Zhong, W.; Hong, Q. L.; Ai, X.; Zhang, C.; Li, F. M.; Li, X. F.; Chen, Y. RhNi bimetallenes with lattice□compressed Rh skin towards ultrastable acidic nitrate electroreduction. *Adv. Mater.* **2024**, 36(23), 2314351.
[42] Zhang, R.; Hong, H.; Liu, X.; Zhang, S.; Li, C.; Cui, H.; Wang, Y.; Liu, J.; Hou, Y.; Li, P. Molecular engineering of a metal□organic polymer for enhanced electrochemical nitrate□to□ammonia conversion and zinc nitrate batteries. *Angew. Chem. Int. Ed.* **2023**, *62* (48), e202309930.

[43] Jiang, H.; Chen, G. F.; Hai, G.; Wang, W.; Liang, Z.; Ding, L. X.; Yuan, Y.; Lu, J.; Antonietti, M.; Wang, H. A nitrogen battery electrode involving eight□electron transfer per nitrogen for energy storage. *Angew. Chem. Int. Ed.* **2023**, *62* (30), e202305695.

[44] Xie, M.; Tang, S.; Li, Z.; Wang, M.; Jin, Z.; Li, P.; Zhan, X.; Zhou, H.; Yu, G. Intermetallic single-atom alloy In–Pd bimetallene for neutral electrosynthesis of ammonia from nitrate. *J. Am. Chem. Soc.* **2023**, *145* (25), 13957-13967.

[45] Zhu, X.; Ma, C.; Wang, Y.-C.; Qu, K.; Song, L.; Wang, J.; Gong, Y.; Liu, X.; Zhang, J.; Lu, Q. Mott–Schottky contact synergistically boosts the electroreduction of nitrate to ammonia under low-nitrate concentration. *Energy Environ. Sci.* **2024**, *17* (8), 2908-2920.

[46] Zhou, L.; Chen, X.; Zhu, S.; You, K.; Wang, Z. J.; Fan, R.; Li, J.; Yuan, Y.; Wang, X.; Wang, J. Two□dimensional Cu plates with steady fluid fields for high□rate nitrate electroreduction to ammonia and efficient Zn□nitrate batteries. *Angew. Chem. Int. Ed* **2024**, *63* (18), e202401924.

[47] Zhou, J.; Xiong, Y.; Sun, M.; Xu, Z.; Wang, Y.; Lu, P.; Liu, F.; Hao, F.; Feng, T.; Ma, Y. Constructing molecule-metal relay catalysis over heterophase metallene for high-performance rechargeable zinc-nitrate/ethanol batteries. *Proc. Natl. Acad. Sci. U.S.A.* **2023**, *120* (50), e2311149120.

[48] Guo, Y.; Zhang, R.; Zhang, S.; Zhao, Y.; Yang, Q.; Huang, Z.; Dong, B.; Zhi, C. Pd doping-weakened intermediate adsorption to promote electrocatalytic nitrate reduction on TiO_2 nanoarrays for ammonia production and energy supply with zinc–nitrate batteries. *Energy Environ. Sci.* **2021**, *14* (7), 3938-3944.

[49] Zhang, R.; Guo, Y.; Zhang, S.; Chen, D.; Zhao, Y.; Huang, Z.; Ma, L.; Li, P.; Yang, Q.; Liang, G. Efficient ammonia electrosynthesis and energy conversion through a zn□nitrate battery by iron doping engineered nickel phosphide catalyst. *Adv. Energy Mater.* **2022**, *12* (13), 2103872.

[50] Lin, H.; Wei, J.; Guo, Y.; Li, Y.; Lu, X.; Zhou, C.; Liu, S.; Li, Y. y. Bi_1□$CuCo_2O_4$ Hollow Carbon Nanofibers Boosts NH3 Production from Electrocatalytic Nitrate Reduction. *Adv. Funct. Mater.* **2024**, 34, 2409696.

[51] Feng, C.; Wu, H.; Shao, J.; Huo, Q.; Hassan, A.; Yang, H.; Hu, Q.; He, C. Sub□Nanometer□Scale Cu_9S_5 Enables Efficiently Electrochemical Nitrate Reduction to Ammonia. *Adv. Energy Mater.* **2024**, 15(8), 2403354.

[52] Yao, Y.; Wei, X.; Zhou, H.; Wei, K.; Kui, B.; Wu, F.; Chen, L.; Wang, W.; Dai, F.; Gao, P. Regulating the d-Band Center of Metal–Organic Frameworks for Efficient Nitrate Reduction Reaction and Zinc-Nitrate Battery. *ACS Catal.* **2024**, *14*(21), 16205-16213.

[53] Ma, C.; Zhang, H.; Xia, J.; Zhu, X.; Qu, K.; Feng, F.; Han, S.; He, C.; Ma, X.; Lin, G. Screening of intermetallic compounds based on intermediate adsorption equilibrium for electrocatalytic nitrate reduction to ammonia. *J. Am. Chem. Soc.* **2024**, *146* (29), 20069-20079.

[54] Zhang, R.; Zhang, S.; Guo, Y.; Li, C.; Liu, J.; Huang, Z.; Zhao, Y.; Li, Y.; Zhi, C. A Zn–nitrite battery as an energy-output electrocatalytic system for high-efficiency ammonia synthesis using carbon-doped cobalt oxide nanotubes. *Energy Environ. Sci.* **2022**, *15*(7), 3024-3032.

[55] Wang, G.; Wang, C.; Tian, X.; Li, Q.; Liu, S.; Zhao, X.; Waterhouse, G. I. N.; Zhao, X.; Lv, X.; Xu, J. Facile construction of CuFe□based metal phosphides for synergistic NO_x^- reduction to NH_3 and Zn□nitrite batteries in electrochemical cell. *Small* **2023**, 20(24), 2311439.

[56] Cui, Z.; Zhao, P.; Wang, H.; Li, C.; Peng, W.; Fan, X.; Liu, J. Molten salts etching strategy construct alloy/MXene heterostructures for efficient ammonia synthesis and energy supply via Zn-nitrite battery. *Appl. Catal. B Environ. Energy* **2024**, 123862.
[57] Zhang, L.; Liang, J.; Wang, Y.; Mou, T.; Lin, Y.; Yue, L.; Li, T.; Liu, Q.; Luo, Y.; Li, N. High□performance electrochemical NO reduction into NH_3 by MoS_2 nanosheet. *Angew. Chem. Int. Ed* **2021**, *60* (48), 25263-25268.
[58] Ren, J. T.; Chen, L.; Wang, H. Y.; Yuan, Z. Y. Aqueous rechargeable Zn–N_2 battery assembled by bifunctional cobalt phosphate nanocrystals-loaded carbon nanosheets for simultaneous NH_3 production and power generation. *ACS Appl. Mater. Interfaces* **2021**, *13* (10), 12106-12117.
[59] Lv, X. W.; Liu, Y.; Wang, Y. S.; Liu, X. L.; Yuan, Z. Y. Encapsulating vanadium nitride nanodots into N, S-codoped graphitized carbon for synergistic electrocatalytic nitrogen reduction and aqueous Zn-N_2 battery. *Appl. Catal. B Environ. Energy* **2021**, *280*, 119434.
[60] Lv, X. W.; Liu, X. L.; Gao, L. J.; Liu, Y. P.; Yuan, Z. Y. Iron-doped titanium dioxide hollow nanospheres for efficient nitrogen fixation and Zn–N_2 aqueous batteries. *J. Mater. Chem. A* **2021**, *9* (7), 4026-4035.
